# Fused in sarcoma regulates DNA replication timing and kinetics

**DOI:** 10.1016/j.jbc.2021.101049

**Published:** 2021-08-08

**Authors:** Weiyan Jia, Sang Hwa Kim, Mark A. Scalf, Peter Tonzi, Robert J. Millikin, William M. Guns, Lu Liu, Adam S. Mastrocola, Lloyd M. Smith, Tony T. Huang, Randal S. Tibbetts

**Affiliations:** 1 Department of Human Oncology, University of Wisconsin School of Medicine and Public Health, Madison, Wisconsin, USA; 2 Department of Chemistry, University of Wisconsin-Madison, Madison, Wisconsin, USA; 3 Department of Biochemistry and Molecular Pharmacology, New York University Langone Health, New York, New York, USA; 4 Department of Biostatistics and Medical Informatics, University of Wisconsin School of Medicine and Public Health, Madison, Wisconsin, USA

**Keywords:** fused in sarcoma (FUS), DNA replication, DNA repair, replication timing, RNA binding protein, amyotrophic lateral sclerosis (ALS), ALS, amyotrophic lateral sclerosis, BrdU, 5-bromo-2′-deoxyuridine, BSA, bovine serum albumin, cDNA, complementary DNA, CF, chromatin fraction, CHOP, CCAAT/enhancer-binding protein homologous protein, CldU, 5-chloro-2′-deoxyuridine, CLM, calicheamicin γ1, CSK, cytoskeleton, DAPI, 4′,6-diamidino-2-phenylindole, DDR, DNA damage response, DSB, double-strand break, EdU, 5-ethynyl-2′-deoxyuridine, ERD, early replication domain, FET, FUS, EWSR1, TAF15, FTD, frontotemporal dementia, FUS, fused in sarcoma, GO, Gene Ontology, HDR, homology-directed repair, HEK293T, human embryonic kidney 293T, HU, hydroxyurea, IP, immunoprecipitation, iPOND, isolation of proteins on nascent DNA, LCD, low-complexity domain, LOF, loss-of-function, LRD, late replication domain, MMC, mitomycin C, MRD, mid replication domain, NHEJ, nonhomologous end joining, ORC, origin recognition complex, PAR, poly(ADP)-ribosyl, PARP, poly(ADP)-ribosyl polymerase, PCNA, proliferating cell nuclear antigen, PI, propidium iodide, pre-RC, prereplication complex, qPCR, quantitative PCR, RD, replication domain, RF, replication fork, RGG, arginine–glycine–glycine repeat, RIPA, radioimmunoprecipitation assay, RT, replication timing, SCAI, suppressor of cancer cell invasion, SSB, single-strand break

## Abstract

Fused in sarcoma (FUS) encodes an RNA-binding protein with diverse roles in transcriptional activation and RNA splicing. While oncogenic fusions of FUS and transcription factor DNA-binding domains are associated with soft tissue sarcomas, dominant mutations in FUS can cause amyotrophic lateral sclerosis. FUS has also been implicated in genome maintenance. However, the underlying mechanisms of its actions in genome stability are unknown. Here, we applied gene editing, functional reconstitution, and integrated proteomics and transcriptomics to illuminate roles for FUS in DNA replication and repair. Consistent with a supportive role in DNA double-strand break repair, FUS-deficient cells exhibited subtle alterations in the recruitment and retention of double-strand break–associated factors, including 53BP1 and BRCA1. *FUS*^*−/−*^ cells also exhibited reduced proliferative potential that correlated with reduced speed of replication fork progression, diminished loading of prereplication complexes, enhanced micronucleus formation, and attenuated expression and splicing of S-phase–associated genes. Finally, FUS-deficient cells exhibited genome-wide alterations in DNA replication timing that were reversed upon re-expression of *FUS* complementary DNA. We also showed that FUS-dependent replication domains were enriched in transcriptionally active chromatin and that FUS was required for the timely replication of transcriptionally active DNA. These findings suggest that alterations in DNA replication kinetics and programming contribute to genome instability and functional defects in FUS-deficient cells.

Fused in sarcoma (FUS, also referred to as translocated in liposarcoma) is a member of the FET (FUS, EWSR1, and TAF15) family of RNA- and DNA-binding proteins that play important roles in transcription and splicing (1, 2). Originally described as an oncogenic fusion to the CCAAT/enhancer-binding protein homologous protein (CHOP) transcription factor in myxoid liposarcoma (3, 4), *FUS* rose to prominence with the discovery that inherited, and *de novo* mutations in its ORF cause dominant forms of amyotrophic lateral sclerosis (ALS) and frontotemporal dementia (FTD) (5, 6, 7). Although the underlying mechanisms are still unclear, the preponderance of ALS/FTD-associated mutations in FUS interferes with its nuclear import and folding, leading to the accumulation of cytosolic FUS aggregates that disrupt cellular function through loss-of-function (LOF) and gain-of function mechanisms impacting protein translation and nuclear transport among other processes (2, 8, 9, 10, 11).

FET proteins share a common domain structure that includes an N-terminal low-complexity domain (LCD), a Gly-rich region, one or more arginine–glycine—glycine repeat (RGG) domain, an RNA recognition motif with RNA- and DNA-binding activity, a zinc-finger domain, and a carboxyl-terminal PY-type nuclear localization signal that interacts with transportin nuclear import receptors that are essential for proper FUS folding (1, 8, 12, 13, 14). The LCD is also of particular interest as it exhibits strong transcriptional coactivation potential *in vitro*, and the fusion of this domain to the CHOP DNA-binding domain drives gene deregulation and oncogenesis in myxoid liposarcoma (1, 15). The LCD also mediates protein–protein interactions and participates in FUS oligomerization and liquid demixing (14, 16, 17, 18, 19) that may be central to its normal roles in transcription and splicing and pathologic roles in ALS/FTD (2).

In addition to their accepted roles in RNA processing, several lines of evidence support a role for the FET proteins in the cellular DNA damage response (DDR). FUS participation in the DDR was first inferred from chromosome instability and mild radiosensitive phenotypes of *FUS*^*−/−*^ mice (20, 21, 22). FET proteins are capable of promoting invasion and pairing of a homologous ssDNA sequence with a dsDNA molecule *in vitro* (22, 23, 24), which suggests a possible role for FET proteins in the D-loop formation step of homology-directed repair (HDR) of DNA double-strand breaks (DSBs). Other studies showed that the FUS LCD is phosphorylated in response to DNA damage by DNA damage–activated protein kinases DNA-PKcs and ATM (17, 25), which are important regulators of the nonhomologous end joining (NHEJ) pathway of DSB repair. Consistent with a direct or an indirect role for FUS in DSB repair, we and others showed that shRNA-mediated depletion of FUS reduced the repair of HDR and NHEJ reporter substrates (26, 27, 28).

A role in the DDR is further suggested by poly(ADP)-ribosyl (PAR) polymerase (PARP)–dependent localization of FUS to sites of microirradiation-induced DNA damage (26, 27, 28). FUS is capable of interacting directly with PAR chains through its RGG domain (26), and the FET proteins are heavily PARylated in response to genotoxic stress (29). Mechanistically, it was reported that FUS mediates the recruitment of histone deacetylase 1, KU70, NBS1, and phosphorylated H2AX (γH2AX), and ATM at sites of DNA damage and that this recruitment pathway as well as FUS-dependent repair was compromised by ALS/FTD-associated mutations (27). It has also been proposed that FUS organizes DSBs in a PARP-dependent manner for their subsequent repair (30); while Wang *et al.* (31) reported that FUS recruits DNA ligase III downstream of PARP activation to repair single-strand breaks (SSBs) and that ALS-associated mutations in FUS disrupt SSB repair activity. Finally, it was recently reported that FUS regulates the response to transcription-associated recombinant DNA damage *via* association with topoisomerase 1 in the nucleolus (32). Despite these studies, the molecular mechanisms linking FUS to the different repair pathways in which it has been implicated remain unclear and the extent to which FUS-dependent RNA processing may contribute to reported DDR phenotypes in FUS-deficient cells is not known.

Here, we probed FUS-dependent genome protection using transcriptomic, proteomic, and functional analysis of *FUS*^*−/−*^ cell lines reconstituted with FUS complementary DNAs (cDNAs). Our findings suggest that FUS plays particularly important roles in DNA replication where it contributes to replicon initiation and coordinates DNA replication timing (RT). These studies provide new insights into FUS-mediated genome protection in mitotically active cells.

## Results

### Generation and phenotypic characterization of *FUS*^*−/−*^ cells

**Figure 1:**
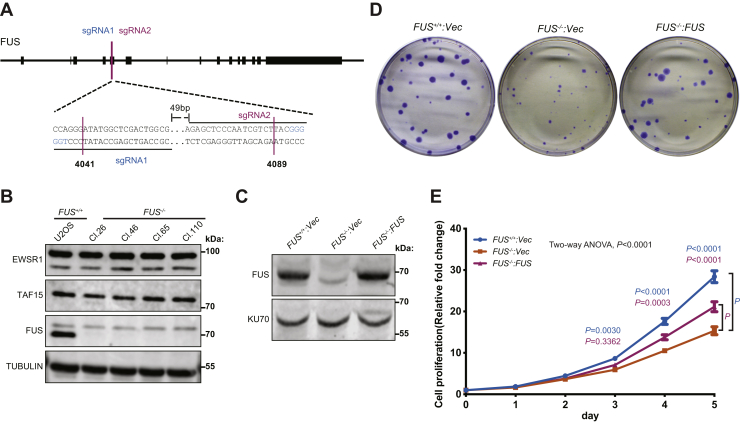
**FUS promotes cell proliferation.***A*, schematic of the FUS gene targeting. Two guide RNAs, sgRNA1 and sgRNA2, were used to target FUS exon 4 (see Experimental procedures section). *B*, expression of FET proteins (FUS, EWSR1, and TAF15) in *FUS*^*−/−*^ clones. *C*, reconstitution of *FUS*^*−/−*^ (Cl.110) with an untagged FUS retroviral vector. The same vector expressing β-glucuronidase (GUS) was introduced as a negative control into *FUS*^*−/−*^ cells. *D*, *FUS*^*−/−*^ cell colonies exhibited reduced growth relative to *FUS*^*+/+*^ and *FUS*^*−/−*^:*FUS* cells. *E*, cell proliferation rates of *FUS*^*+/+*^, *FUS*^*−/−*^, and *FUS*^*−/−*^: FUS U-2 OS cells. Three biological replicates were used. The bars represent mean ± SE. The two-way ANOVA test was performed, and the *p* values shown on plot are adjusted *p* values by Tukey's multiple comparisons test. FUS, fused in sarcoma.

To discern roles of FUS in genome protection, we disrupted *FUS* gene loci in U-2 OS osteosarcoma cells using CRISPR–CAS9 followed by genetic reconstitution with a retroviral vector encoding the untagged FUS ORF (see Experimental procedures section). To ensure rigorous results, we studied multiple *FUS*^*−/−*^ clones and selected a reconstituted *FUS*^*−/−*^*:FUS* line with physiological levels of FUS expression (Fig. 1, *A*–*C*). Notably, protein levels of TAF15 and EWSR1 were not upregulated in *FUS*^*−/−*^ U-2 OS cells, diminishing concerns about functional compensation.

FUS knockdown cells displayed mild IR sensitivity and modest defects in the repair of NHEJ and HDR reporter substrates (26), whereas a second study reported that FUS knockdown suppressed γH2AX and 53BP1 focus formation (27). We assessed time courses of γH2AX and 53BP1 accumulation and dissolution at IR-induced foci in *FUS*^*+/+*^, *FUS*^*−/−*^, and *FUS*^*−/−*^*:FUS* U-2 OS cells exposed to 2 Gy IR. The initial recruitment of 53BP1 to IR-induced nuclear foci was enhanced in *FUS*^*−/−*^ cell lines, and 53BP1 foci persisted longer in *FUS*^*−/−*^ cells relative to *FUS*^*+/+*^ cells (Fig. S1, *A* and *C*), and this was rescued in *FUS*^*−/−*^*:FUS* U-2 OS cells. The enhanced and prolonged accumulation of 53BP1 at IR-induced foci may reflect persistent DSBs. While no obvious γH2AX focus formation/dissociation defect was observed between *FUS*^*−/−*^ and *FUS*^*+/+*^ cells, we observed reduced γH2AX focus formation in *FUS*^*−/−*^*:FUS* U-2 OS cells relative to *FUS*^*−/−*^ cells (Fig. S1, *B* and *C*). Although the reason for discrepant findings between *FUS*^*+/+*^ and *FUS*^*−/−*^*:FUS* U-2 OS cells is unclear, it may reflect slightly increased FUS expression levels in *FUS*^*−/−*^*:FUS* U-2 OS cells relative to *FUS*^*+/+*^ cells (Fig. 1*C*).

We also investigated recruitment of the critical HDR factor, BRCA1. On a per-cell basis, the number of BRCA1 foci was comparable between *FUS*^*−/−*^, *FUS*^*+/+*^, and *FUS*^*−/−*^*:FUS* cells, suggesting FUS is not an essential component of the BRCA1 recruitment pathway (Fig. S2, *A* and *C*). On the other hand, the frequency of cells displaying IR-induced BRCA1 foci was significantly reduced in *FUS*^*−/−*^ cells, and this was corrected by FUS reexpression (Fig. S2, *B* and *C*). Reduced BRCA1 focus formation was also seen in H460 cells stably transduced with FUS shRNA but not cells transduced with TAF15 or EWSR1 shRNA (Fig. S2, *D* and *E*), indicating a selective role for FUS. Because BRCA1 focus formation is largely restricted to S/G2 phase, these findings may indicate perturbed S-phase dynamics in FUS deficiency (see later). Despite the changes in 53BP1 and BRCA1 recruitment to IR-induced foci, *FUS*^*−/−*^ cells did not exhibit significant hypersensitivity to mechanistically distinct genotoxins, including hydroxyurea (HU, replication stress), mitomycin C (MMC, DNA crosslinker), camptothecin (top1 inhibitor), and calicheamicin γ1 (CLM, radiomimetic). In fact, *FUS*^*−/−*^*:FUS* cells were slightly more resistant than *FUS*^*+/+*^ cells to MMC and CLM (Fig. S3). These findings suggest that FUS fulfills supportive rather than essential roles in DSB repair.

### *FUS*^*−/−*^ cells exhibit defects in DNA replication

*FUS*^*−/−*^ U-2 OS cells exhibited reduced colony outgrowth and proliferative potential that was corrected by FUS reexpression (Fig. 1, *D* and *E*). Reduced proliferative capacity was observed in multiple *FUS*^*−/−*^ U-2 OS clones as well as FUS-deficient NCI-H460 lung adenocarcinoma cells (Fig. S4, *A* and *B*). Finally, *FUS*^*−/−*^ U-2 OS reconstituted with a FUS construct lacking the N-terminal LCD exhibited reduced colony growth rates relative to *FUS*^*−/−*^*:FUS* cells (Fig. S4, *C* and *D*). This finding implies that biochemical activities associated with the LCD, including transcriptional activation (15, 33) and phase separation/oligomerization (19, 34), contribute to its replication-associated functions.

**Figure 2:**
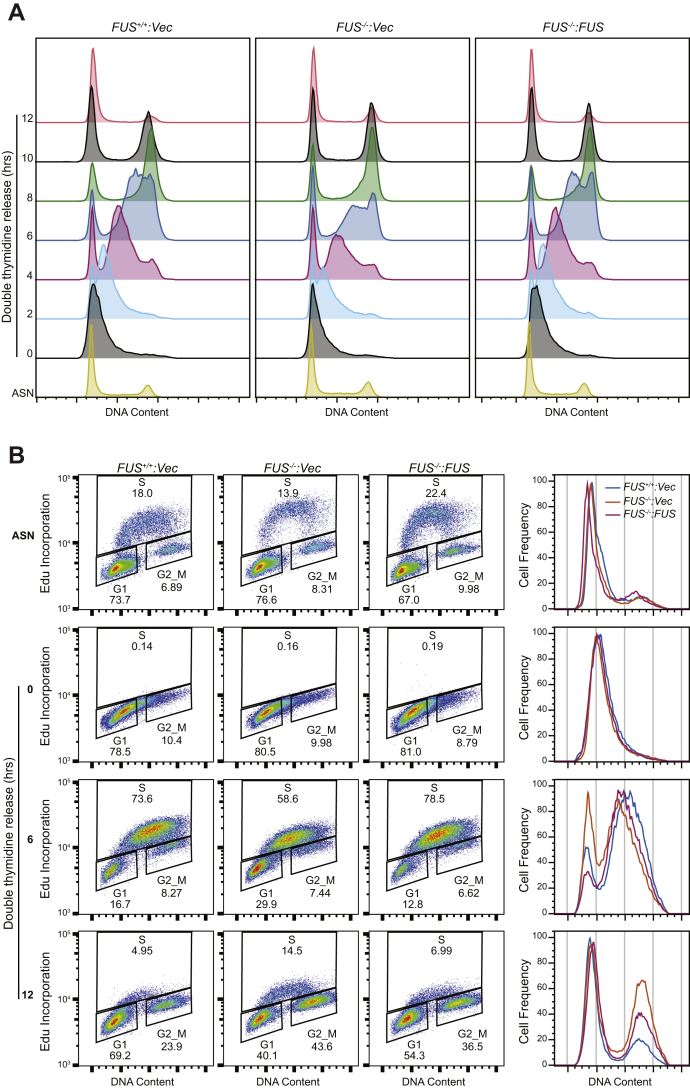
**FUS is required for S-phase progression.***A*, DNA replication progression was analyzed by PI staining and flow cytometry. Cells were synchronized to G_1_/S boundary by double thymidine block and released into fresh growth medium for the indicated times and stained with PI for cell cycle analysis. *B*, DNA progression was monitored by EdU incorporation under the same conditions as in (*A*). Additional time points are presented in Fig. S5*D*. EdU, 5-ethynyl-2′-deoxyuridine; FUS, fused in sarcoma; PI, propidium iodide.

Following synchronous release from G_1_/S phase arrest, *FUS*^*−/−*^ cells exhibited reduced reentry and progression through S phase, which was particularly pronounced at the 6 h time point (Fig. 2*A*, Fig. S5, *A–C*). The S-phase delay of *FUS*^*−/−*^ cells was further revealed through 5-ethynyl-2′-deoxyuridine (EdU) incorporation experiments. Specifically, *FUS*^*−/−*^ cells exhibited reduced S-phase entry 6 h following release from a double thymidine block and accumulated in G_2_/M to a lesser degree than *FUS*^*+/+*^ or *FUS*^*−/−*^:*FUS* cells 12 h following release (Fig. 2*B*; see Fig. S5*D* for additional time points). These experiments also revealed slightly reduced levels of EdU incorporation in asynchronously growing *FUS*^*−/−*^ cells relative to *FUS*^*+/+*^ or *FUS*^*−/−*^:*FUS* cells (Fig. 2*B*).

**Figure 3:**
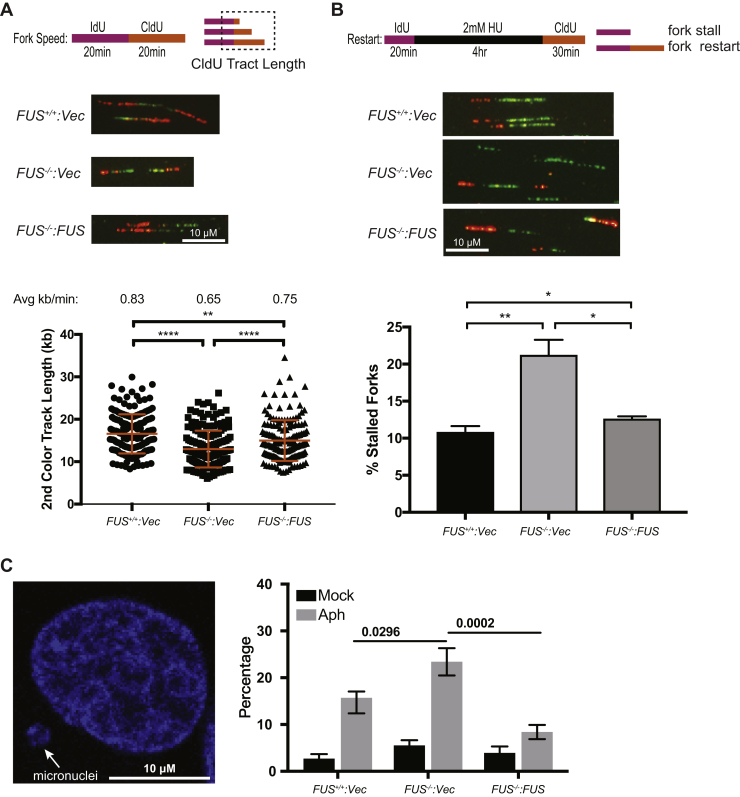
**FUS deficiency leads to genomic instability and replication stress.***A*, replication fork speed is reduced in *FUS*^*−/−*^ cells. The second pulse (CIdU) was used for measurement of track length, which was converted to micrometers using a 1 μm = 2.59 kb conversion factor. The average fork length was divided by 20 min to derive replication speed. *B*, replication fork restart was measured as shown in the schematic. Percentages of fork restart (percent of stalled forks) in HU-treated cells are shown. *A* and *B*, the representative DNA fiber images were included. Data are mean ± SD (n = 3). *p* Values were calculated using a *t* test with Welch's correction. ∗*p* < 0.05, ∗∗*p* < 0.01, ∗∗∗*p* < 0.001, and ∗∗∗∗*p* < 0.0001. *C*, *FUS*^*+/+*^, *FUS*^*−/−*^, and *FUS*^*−/−*^*:FUS* U-2 OS cells were treated with or without 0.2 μM aphidicolin (Aph) for 24 h, fixed, and stained with DAPI for micronucleus counting. *p* Values were calculated by two-way ANOVA test. Data are means ± SE (n = 3 biological replicates). More than 250 cells for each sample in each biological replicate were counted. CIdU, 5-chloro-2′-deoxyuridine; DAPI, 4′,6-diamidino-2-phenylindole; FUS, fused in sarcoma; HU, hydroxyurea; ns, no significance.

To ascertain impacts of FUS deficiency on replication fork (RF) dynamics, we performed DNA fiber analysis (35), on *FUS*^*−/−*^, *FUS*^*+/+*^, *FUS*^*−/−*^*:FUS* cells sequentially labeled with 5-iodo-2′-deoxyuridine and 5-chloro-2′-deoxyuridine (CldU). *FUS*^*−/−*^ cells exhibited significant reductions in CldU track lengths indicative of reduced DNA replication rate (Fig. 3*A*). *FUS*^*−/−*^ cells also showed delayed RF restart following release from a transient HU block (Fig. 3*B*). Both replication velocity and replication restart phenotypes were rescued by FUS reexpression.

Because a reduced rate of DNA replication can lead to micronucleus formation and genomic instability (36), we measured micronuclei in *FUS*^*+/+*^, *FUS*^*−/−*^, and *FUS*^*−/−*^*:FUS* U-2 OS cells treated with a low dose of the DNA polymerase alpha inhibitor aphidicolin. *FUS*^*−/−*^ cells exhibited increased rates of micronucleus formation relative to *FUS*^*+/+*^ and *FUS*^*−/−*^*:FUS* U-2 OS cells (Fig. 3*C*), suggesting that FUS enhances genome stability under replication stress.

### Reduced S-phase gene expression in FUS-deficient cells

**Figure 4:**
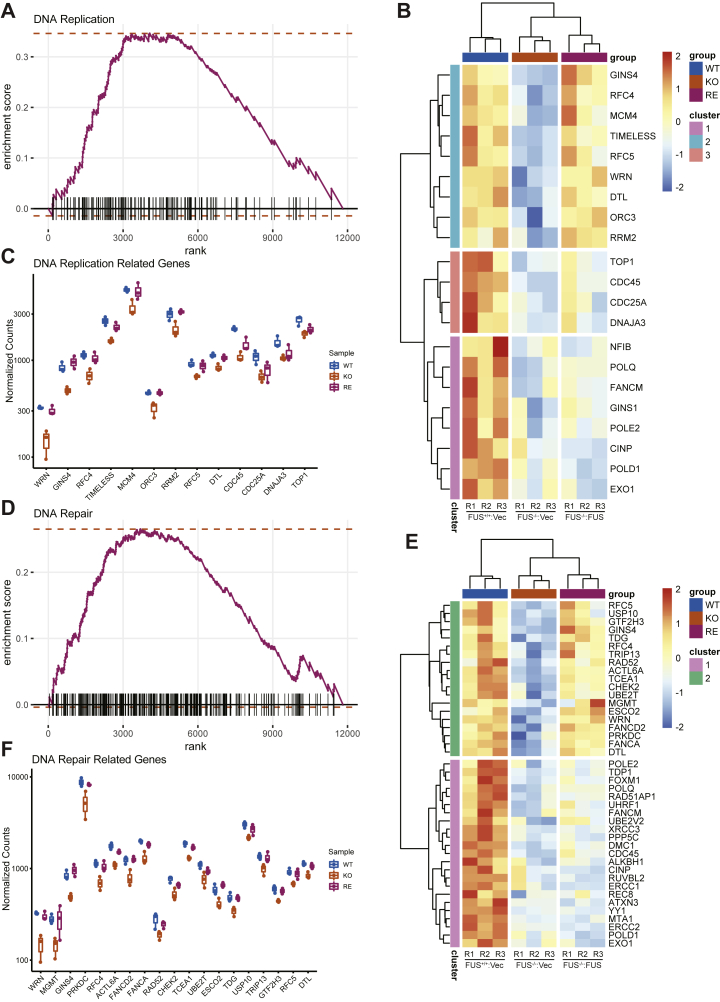
**Reduced expression of replication-associated genes in FUS-deficient cells.***A*, enrichment plot of DNA replication pathway from GSEA using GO gene sets (biological process) in Table S2. *B*, heat map of differentially expressed DNA replication genes. Genes were clustered to three groups based on ward.D2 method. *C*, normalized RNA-Seq counts of cluster 2 genes involved in the DNA replication pathway. *D*, enrichment plot of DNA repair pathway from GSEA using GO gene sets (biological process) in Table S2. *E*, heat map of the leading gene list of DNA repair pathway showed significant change in all samples. Genes were clustered into two groups based on ward.D2 method. *F*, DNA repair–related gene expressions in cluster 1 were shown in normalized counts from RNA-Seq results. FUS, fused in sarcoma; GO, Gene Ontology; GSEA, gene set enrichment analysis.

We performed RNA-Seq to establish gene expression correlates for DNA replication defects of FUS-deficient cells (Table S1). We identified 710 genes that were differentially expressed between *FUS*^*+/+*^ and *FUS*^*−/−*^ cells that were corrected by FUS reexpression (Fig. S6, *A* and *B* and Table S3). Gene set enrichment analysis revealed that cell cycle, DNA repair, and DNA replication processes were downregulated, whereas immunomodulatory pathways were upregulated in *FUS*^*−/−*^ cells (Fig. S6*C* and Fig. 4, *A* and *D*). DNA replication–associated genes that were downregulated in *FUS*^*−/−*^ cells included *GINS4*, *MCM4*, *RFC3*, *RCF4*, and *TIMELESS* (Fig. 4, *B* and *C*). DNA repair–related genes, including *WRN*, *PRKDC*, *FANCD2*, *FANCA*, and *RAD52*, were also downregulated in *FUS*^*−/−*^ cells (Fig. 4, *E* and *F*). Interestingly, the NHEJ factor *53BP1* was upregulated in *FUS*^*−/−*^ cells (Fig. S6*E*). A subset of gene expression changes evident in RNA-Seq data were confirmed by quantitative PCR (qPCR; Fig. S6, *D* and *E*). Downregulation of S-phase genes may contribute to reduced proliferative potential of *FUS*^*−/−*^ cells and/or may be a downstream consequence of DNA replication abnormalities.

We next considered the possibility that FUS regulates the alternative splicing of suites of genes involved in DNA replication and repair. We thus compared alternative splicing patterns between *FUS*^*+/+*^, *FUS*^*−/−*^, and *FUS*^*−/−*^*:FUS* cells. We identified 434 splicing events that differed between *FUS*^*−/−*^ and *FUS*^*+/+*^ cells including alternative 5′ splice site selection, exon skipping events, altered 3′ splice site selection, and intron retention (Fig. S6*F* and Table S6). While DNA repair was not overrepresented in Gene Ontology (GO) terms, we nonetheless identified a handful of genes with annotated roles in DNA repair and replication that exhibited FUS-dependent splicing changes (Fig. S6, *G* and *H*). For instance, both origin recognition complex 3 (ORC3) and suppressor of cancer cell invasion (SCAI) saw increased inclusion of poison cassette exons predicted to terminate their respective ORFs and/or promote mRNA degradation *via* nonsense-mediated mRNA decay (Fig. S6, *I*–*N*). ORC3 is a component of the eukaryotic origin recognition complex, whereas SCAI is a negative regulator of the NHEJ factor RIF1 and has been implicated in restricting chromatin access to DSB factors (37, 38). Although functional implications are unclear, the inclusion of poison cassette exons may reduce ORC3 and/or SCAI gene dosage. We also identified an alternative cassette exon in the ubiquitin E3 ligase TRIP12 that was increased in *FUS*^*−/−*^ U-2 OS cells relative to controls and rescued by FUS reexpression (Fig. S6, *O*–*Q*). TRIP12 has been implicated in the ubiquitylation of the p53 regulator ARF and RNF168 (39, 40), and its FUS-dependent alternative splicing may alter its activity toward ARF, RNF168, or other targets.

### FUS regulates prereplication complex loading and associates with DNA replication factors

**Figure 5:**
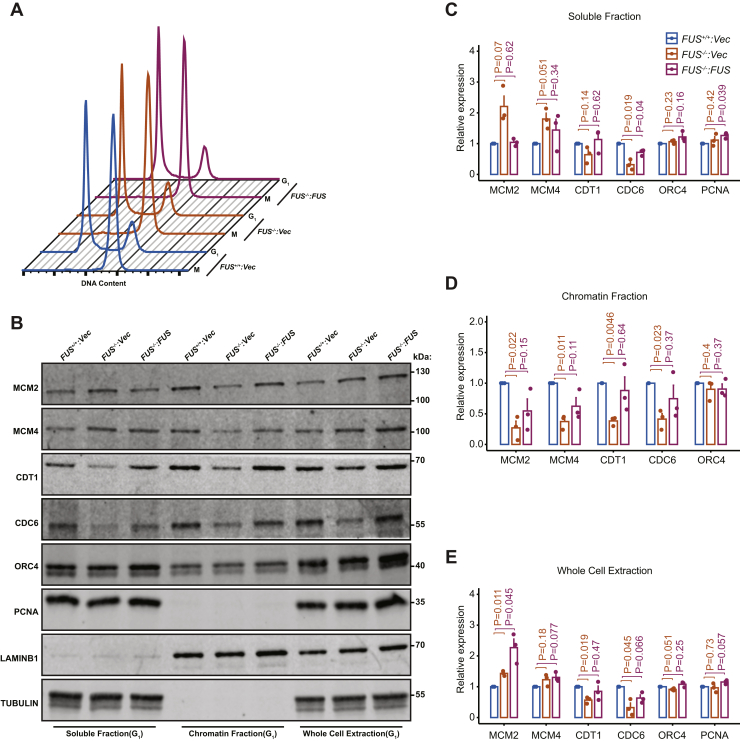
**FUS is required for efficient prereplication complex (pre-RC) loading.***A*, cell cycle profiles of *FUS*^*+/+*^, *FUS*^*−/−*^, and *FUS*^*−/−*^*:FUS* U-2 OS cells that were synchronized in early M phase with nocodazole (0.1 μg/ml for 16 h) and then harvested or released into G_1_ phase for 5 h. *B*, chromatin loading of ORC and pre-RC proteins in *FUS*^*+/+*^, *FUS*^*−/−*^, and *FUS*^*−/−*^*:FUS* U-2 OS cells. G_1_ fractions were immunoblotted with the indicated antibodies. *C*–*E*, quantification of Western blotting results for soluble fractions (SFs, panel *C*), chromatin fractions (CFs, panel *D*), and whole-cell extracts (WCE, panel *E*) shown in panel *B*. Three independent biological replicates were used for the quantification. Data are means ± SE (n = 3 biological replicates). *p* Values were calculated by Student's *t* test for comparison between two samples. The expression of proteins in CF was normalized to lamin B1, SF were normalized to tubulin, and WCE were normalized with mean of lamin B1 and tubulin. FUS, fused in sarcoma; ORC, origin recognition complex.

Given their reduced DNA replication rate, we investigated whether *FUS*^*−/−*^ cells exhibited defects in the chromatin loading of replication licensing factors, including the ORC, CDC6, CDT1, and the MCM replicative helicase (41). Mitotically arrested *FUS*^*+/+*^, *FUS*^*−/−*^, and *FUS*^*−/−*^*:FUS* cells were released into early G_1_ phase, and soluble and chromatin fractions (CFs) were analyzed by immunoblotting. FUS-deficient cells showed normal cell progression from G_2_/M to G_1_ phase and unchanged ORC loading onto chromatin in G_1_ (Fig. 5, *A*–*D*). By contrast, both total abundance and chromatin loading of CDC6 and CDT1 was significantly decreased in *FUS*^*−/−*^ cells and rescued by FUS reexpression (Fig. 5, *B*–*E*). There was a corresponding decrease in CDC6-dependent chromatin loading of MCM2 and MCM4 in FUS-deficient U-2 OS cells (Fig. 5, *B*–*E*). Collectively, these results revealed that FUS facilitates ORC-dependent recruitment of prereplication complex (pre-RC) factors to replication origins.

**Figure 6:**
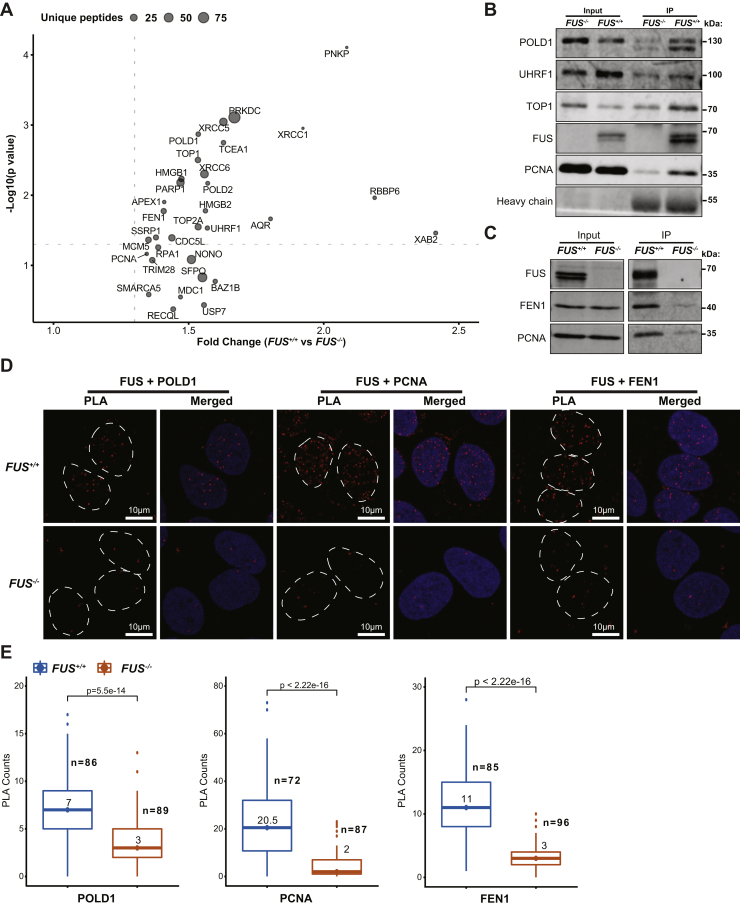
**FUS interacts with DNA repair and DNA replication factors.***A*, FUS-interacting proteins were identified by crosslinking chromatin immunoprecipitation (IP) and analyzed by MS. The results are combination of three biological replicates quantified by nonisotopic spectral peptide counting. The data shown are DNA repair and DNA replication pathway–related interactions based on GSEA (full list is shown in Fig. S6*A*). The unique peptides are summarized from the raw data of the three replicates. The *gray dotted lines* are 1.3 of fold change and 0.05 of *p* value. *B*, co-IP of FUS with POLD1, UHRF1, TOP1, and PCNA in unsynchronized cells. *C*, co-IP of FUS with FEN1 and PCNA in synchronized S-phase cells. *D*, *in situ* proximity ligation assay (PLA) was employed to verify the interactions between FUS and POLD1, PCNA, and FEN1. Nuclear regions were cycled by *dashed lines* in PLA red channel based on DAPI signal. *E*, quantification results of PLA signal in (*D*). The values are median of PLA foci in each sample. *p* Values were calculated by Wilcoxon test method. DAPI, 4′,6-diamidino-2-phenylindole; FUS, fused in sarcoma; GSEA, gene set enrichment analysis.

Reasoning that FUS may play direct roles in pre-RC loading and or DNA repair, we performed quantitative proteomic analysis of FUS complexes using a chromatin immunoprecipitation (IP) procedure in which endogenous FUS–chromatin complexes were digested with nuclease prior to IP with α-FUS antibodies and analysis by quantitative MS (42). The same chromatin IP procedure was carried out using *FUS*^*−/−*^ cells as a negative control. Gene set enrichment analysis using all identified FUS interactants revealed RNA processing, DNA repair, and DNA replication as functional processes that were statistically overrepresented in the dataset of FUS-interacting proteins (Fig. S7*A*, Tables S4 and S5). The abundance of RNA-binding proteins in FUS complexes is consistent with other published studies (43, 44). Nucleotide excision repair and DNA strand elongation were among the most significantly enriched pathways within the DNA replication/repair gene sets (Fig. S7, *B* and *C*). We plotted those proteins within DNA repair and replication GO terms that showed a nominal 1.3-fold enrichment in IPs from *FUS*^*+/+*^ cells relative to *FUS*^*−/−*^ cells (Fig. 6*A*). Proteins of interest include DSB repair factors (DNA-PK, Ku70, Ku80, and PNKP), single-strand break repair/base excision repair proteins (PARP1, FEN1, PNKP, and APEX1), DNA replication factors (DNA polymerase δ [POLδ or POLD1], proliferating cell nuclear antigen [PCNA], and UHRF1), and topoisomerases (TOP1 and TOP2α). The presence of single-strand break repair/base excision repair factors, including PARP, is consistent with the ability of FUS to bind to PAR chains (26), whereas the presence of POLδ but not POLε in FUS IPs is interesting given their participation in lagging strand and leading strand DNA synthesis, respectively (45). We carried out validation co-IP assays to confirm that endogenous FUS interacted with TOP1, PCNA, POLδ1, and FEN1 in unsynchronized (Fig. 6*B*) or synchronized S phase cells (Fig. 6*C*) and further validated association between FUS and POLδ1, PCNA, and FEN1 in proximity ligation assays (Fig. 6, *D* and *E*).

The replication defects in *FUS*^*−/−*^ cells and interaction with DNA replication factors raised the possibility that FUS directly participates in DNA replication. To investigate this possibility, we carried out an isolation of proteins on nascent DNA (iPOND) assay that measures the association of proteins with nascently synthesized DNA (46). Human embryonic kidney 293T (HEK293T) cells were pulse labeled with EdU and then chased with thymidine in the absence or the presence of 2 mM HU prior to formaldehyde crosslinking and isolation of EdU–protein complexes. As expected, the abundance of the PCNA sliding clamp in EdU-labeled complexes decreased during the thymidine chase period as the replisome advanced beyond the region of nascent EdU-labeled DNA (Fig. S8). Although FUS was also observed in EdU-labeled DNA, its abundance was slightly increased following thymidine chase, as was histone H3 (Fig. S8). A similar iPOND labeling pattern has been described for DNA-binding proteins such as HMGA1 and LaminB1 that maintain high-order chromatin (47). This result suggests that FUS is proximal to replication factors on chromatin but does not translocate with the active replisome.

### FUS regulates DNA RT

**Figure 7:**
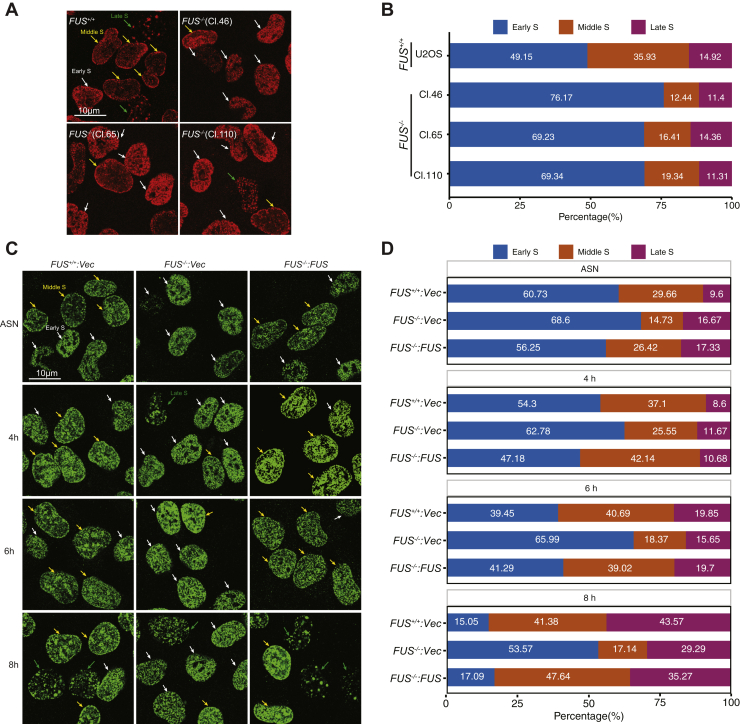
**FUS regulates DNA replication timing (RT).***A*, asynchronous U-2 OS cells and three *FUS*^*−/−*^ clones (Cl. 46, Cl. 65, and Cl. 110) were pulse labeled with EdU for 20 min and scored for the presence of early, mid, or late EdU staining patterns. *B*, quantification analysis of cell numbers of each S-phase patterns in (*A*) and the percentages were calculated in each sample. *C*, cells were synchronized with double thymidine and then released into S phase for indicated times. Cells were then pulse labeled with BrdU, stained, and imaged by confocal microscopy. *D*, quantification results of samples using a minimum 100 cells per sample (*C*). BrdU, 5-bromo-2′-deoxyuridine; EdU, 5-ethynyl-2′-deoxyuridine; FUS, fused in sarcoma.

Chromosomal replication is stochastically initiated from origins that fire with characteristic heritable timing (41). RT can be qualitatively evaluated according to the pattern 5-bromo-2′-deoxyuridine (BrdU) or EdU incorporation following synchronized release from a double thymidine block (48). Early S-phase cells exhibit a uniform EdU incorporation pattern (Fig. 7*A*, *white arrows*); middle S-phase cells exhibit perinuclear and perinucleolar EdU incorporation (Fig. 7*A*, *yellow arrows*); and late S-phase cells exhibit large puncta of EdU incorporation (Fig. 7*A*, *green arrows*). Origins with shared firing kinetics are topologically organized into chromatin subdomains in a process that requires RIF1 (49, 50, 51, 52, 53, 54); however, few other timing regulators have been identified.

We noted that the frequency of mid-S phase staining patterns was reduced ∼50% in *FUS*^*−/−*^ cells relative to *FUS*^*+/+*^ cells (Fig. 7, *A* and *B*), suggesting a potential RT defect. To rule out the apparent defect was not because of delayed S-phase entry of *FUS*^*−/−*^ cells, we carried out a time course analysis of *FUS*^*+/+*^, *FUS*^*−/−*^, and *FUS*^*−/−*^*:FUS* cells released from thymidine block for 4, 6, or 8 h. *FUS*^*−/−*^ cells exhibited reduced frequencies of the mid-S-phase staining pattern at all three time points, even though the frequency of late S-phase patterns more than doubled from 4 to 8 h (Fig. 7, *C* and *D*). Importantly, FUS reexpression largely reversed the mid-S phase RT defect of *FUS*^*−/−*^ cells (Fig. 7, *C* and *D*). From this, we conclude that FUS-deficient cells harbor RT defects that cannot be solely attributed to reduced rates of replication.

**Figure 8:**
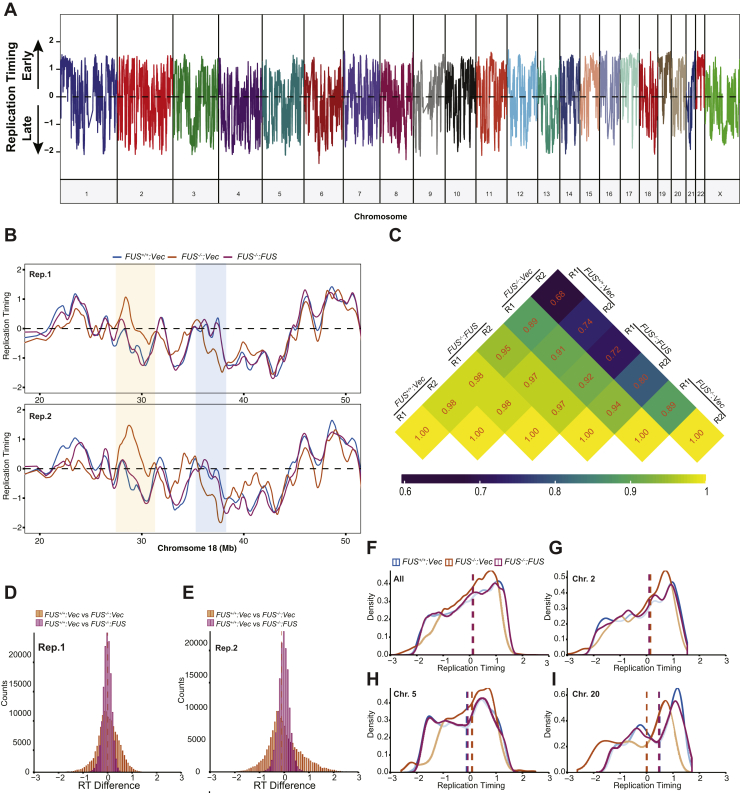
**FUS influences genome-wide RT.***A*, whole genome-wide replication timing profile of U-2 OS cells. The RT was calculated based on copy number variations between S- and G_1_-phase cells (S/G_1_ ratio). The signal was normalized by *Z* score and smoothed by Loess smoothing. *B*, representative RT profiles of *FUS*^*+/+*^, *FUS*^*−/−*^, and *FUS*^*−/−*^*:FUS* cells across two biological replicates. Regions of RT switching between *FUS*^*+/+*^ and *FUS*^*−/−*^ are highlighted. *C*, correlation of RT between two biological replicates by Pearson's method. The smoothed RT values were used for the correlation matrix. *D* and *E*, genome-wide distribution of RT scores when comparing *FUS*^*+/+*^ to *FUS*^*−/−*^ or *FUS*^*+/+*^*versus FUS*^*−/−*^*:FUS* in two biological replicates. The bin sizes are 50 and 100 for Replicate1 (Rep. 1) and Replicate2 (Rep. 2), respectively. *F*–*I*, the RT density distribution for Rep. 2 was analyzed across all chromosomes (*F*), Chr.2 (*G*), Chr.5 (*H*), and Chr.20 (*I*). The RT density distribution for Rep. 1 is shown in Fig. S9, *A*–*D*. The *dashed lines* are the median of each sample. The Loess smoothed data were used for analysis. FUS, fused in sarcoma; RT, replication timing.

To follow up on the EdU labeling studies, we measured genome-wide RT in *FUS*^*+/+*^, *FUS*^*−/−*^, and *FUS*^*−/−*^*:FUS* U-2 OS cells using a Sort-Seq workflow (55) in which propidium iodide (PI)–stained *FUS*^*−/−*^, *FUS*^*+/+*^, and *FUS*^*−/−*^: FUS U-2 OS cells were sorted into G_1_- and S-phase fractions prior to genomic DNA isolation and deep sequencing (see Experimental procedures section). The read ratios between S- and G1-phase cells were used to establish relative DNA copy number between samples, with a higher ratio reflecting earlier replication (Fig. 8*A*). Using a fixed-window method of read binning, we found that *FUS*^*−/−*^ cells exhibited widespread changes in RT relative to *FUS*^*+/+*^ and *FUS*^*−/−*^*:FUS* cells that was consistent across two biological replicates (Fig. 8, *B* and *C*). FUS deficiency impacted RT bidirectionally and was highly chromosome and position dependent. For example, within the same 30 Mb interval of Chr18, *FUS*^*−/−*^ cells exhibited advanced RT (Fig. 8*B*, *tan shading*) and delayed RT (Fig. 8*B*, *blue shading*).

Genome-wide bidirectional RT switches were further confirmed by RT distribution differences between *FUS*^*+/+*^ and *FUS*^*−/−*^ cells in both biological replicates (Fig. 8, *D* and *E*). Although the RT distribution of *FUS*^*−/−*^ cells skewed slightly earlier than *FUS*^*+/+*^ cells when examined across all chromosomes (Fig. 8*F* [replicate 2] and Fig. S9*A* [replicate 1]), RT directional changes were highly chromosome dependent. For example, while Chr2 did not show significant RT distribution differences between *FUS*^*+/+*^ and *FUS*^*−/−*^ cells, the RT distributions of Chr5 an Chr20 skewed early and late, respectively, in *FUS*^*−/−*^ cells relative to *FUS*^*+/+*^ cells (Fig. 8, *G*–*I* [replicate 2] and Fig. S9, *B*–*D* [replicate 1]). In summary, our data indicate that FUS influences genome-wide RT in a chromosomal context-dependent manner.

### Characterization of FUS-dependent replication domains

**Figure 9:**
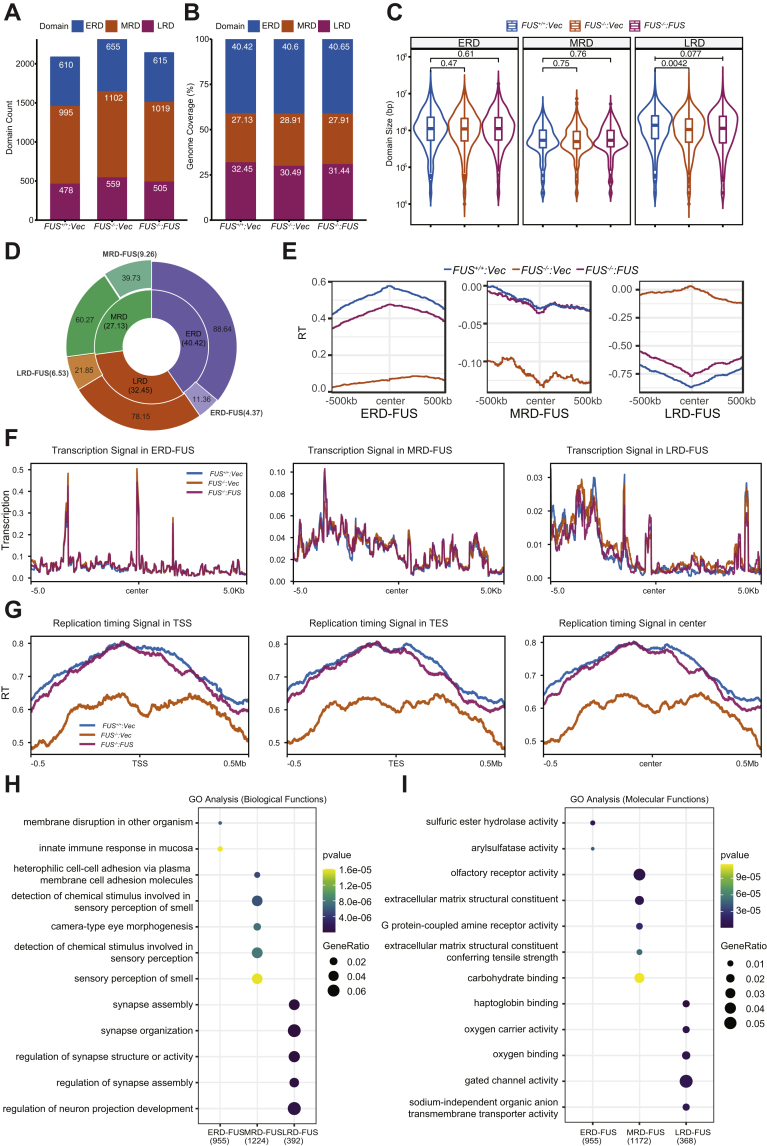
**Characterization of FUS-dependent replication domains (RDs).***A*, RT profiles were segmented into three states by nonsupervised package Segway as early RD (ERD), middle RD (MRD), and late RD (LRD). The domain numbers in each sample were plotted and labeled. The two biological replicates were merged for RD segmentation. *B*, percentages of genome coverage of RDs in each sample were calculated based on the segmentation. The values are percentages of each domain. *C*, the same RT domain sizes are compared among all the samples. The Student's *t* test was used for determination of significance. *D*, doughnut pie chart of FUS-dependent RD coverage. The percentage of each RD (ERD, MRD, and LRD; *center pie*) that is altered by FUS deficiency (FUS-dependent RDs) is shown in the *outside layer*, and the total percentage of each FUS-dependent RDs (ERD-FUS, MRD-FUS, and LRD-FUS) are calculated and shown in *parentheses*. The percentage was calculated based on the genome coverage. *E*, RT signal enrichment analysis of FUS-dependent replication domains in the samples. The average domain size is ∼10^6^ bp *C*, and ∼0.5 × 10^6^ bp flanking the midpoint was used for signal enrichment. Heat map results of RT signal enrichment of changing ERD, MRD, and LRD in all individual samples were shown in Fig. S9, *E*–*G*. *F*, transcription signal in the centered FUS-dependent RDs. Transcription signal was normalized with CPM by STAR. *G*, RT signal enrichment around TSS, TES, and center of FUS-regulated gene regions across a ±0.5 Mb window. RT signal was calculated by log2 ratio of S/G_1_ samples in 20 kb bin after CPM normalization and followed with *Z* score normalization. Only FUS-regulated genes (listed in Table S3) annotation was used. *H*, Gene Ontology (GO) enrichment in biological function level of FUS-dependent RDs. The FUS-dependent RDs were extended 3000 bases in both ends, and then, the gene list under the extended FUS-dependent RDs was extracted and used for GO analysis. *I*, GO analysis in molecular function level of extended FUS-dependent RDs. FUS, fused in sarcoma; RT, replication timing; TES, transcription end site; TSS, transcription start site.

We next used the unsupervised Segway deep learning tool (56, 57, 58) to *de novo* segment replication domains (RDs) in *FUS*^*+/+*^, *FUS*^*−/−*^, and *FUS*^*−/−*^*:FUS* cells (see Experimental procedures section). Three nonoverlapping contiguous segments were used to assign RT profiles into early replication domains (ERDs); late replication domains (LRDs), and mid replication domains (MRDs) spanning the transition between the early and late zone (Fig. 9*A*). Genomic coverage of all three types of RDs did not significantly change between *FUS*^*−/−*^ cells relative to *FUS*^*+/+*^ cells (Fig. 9*B*). However, the average size of LRDs was significantly decreased in *FUS*^*−/−*^ cells (Fig. 9*C*). To determine which fraction of ERDs, MRDs, and LRDs were dependent on FUS, overlapping RDs in *FUS*^*+/+*^ and *FUS*^*−/−*^*:FUS* cells were intersected and then subtracted from corresponding RDs in *FUS*^*−/−*^ cells using bedtools. This analysis revealed that 11.36%, 39.73%, and 21.85% of total ERDs, MRDs, and LRDs, respectively, were FUS dependent (Fig. 9*D*), and we refer to these regions as ERD-FUS, MRD-FUS, and LRD-FUS (Fig. 9*D*). ERD-FUS, MRD-FUS, and LRD-FUS represented 4.37%, 9.26%, and 6.53% of whole genome sequence, respectively, and, in total, approximately 20% of the U-2 OS genome exhibited FUS-dependent RT. Finally, RT signals of FUS-associated RDs were centered, and the distribution and heat map analysis were performed and showed they were correctly identified (Fig. 9*E* and Fig. S9, *E*–*G*).

Consistent with earlier studies (59, 60, 61), a positive correlation between gene activation and RT was found, with ERDs exhibiting active gene expression and LRDs exhibiting repressed gene expression (Fig. 9*F* and Fig. S9*H*). However, overall transcription signals were comparable between *FUS*^*−/−*^ cells and *FUS*^*+/+*^ cells across ERD-FUS, MRD-FUS, and LRD-FUS domains (Fig. 9*F*). We next explored whether RT was changed proximal to genes showing FUS-dependent regulation by RNA-Seq. We found RT of annotated gene regions was delayed in *FUS*^*−/−*^ cells relative to *FUS*^*+/+*^ and *FUS*^*−/−*^*:FUS* cells (Fig. 9*G*). This pattern of delayed timing was observed across the entirety of the gene, including the transcription start site, coding sequence, and termination site, and was observed for both upregulated and downregulated genes. To determine whether delayed RT was restricted to those genes regulated by FUS, we compared relative RT across all annotated genes. As shown in Fig. S9, *I* and *J*, a similar pattern of delayed RT was observed in *FUS*^*−/−*^ cells relative to *FUS*^*+/+*^ and *FUS*^*−/−*^*:FUS* cells. These results imply that FUS plays a particularly important role in the early replication of transcriptionally active chromatin.

Finally, we examined FUS-dependent RDs for gene functional enrichment. Surprisingly, given the non-neuronal nature of U-2 OS cells, we found that LRD-FUS were highly enriched in nervous system development–related genes and, more specifically, genes encoding ion-gated channels (Fig. 9, *H* and *I* and Fig. S10, *A* and *B*). We further explored the transcription of genes in the top ten enriched GO terms in FUS-dependent LRDs (LRD-FUS) (Fig. S10, *A* and *C*). Despite being enriched in LRD-FUS, only ten of 55 genes showed significant expression differences between *FUS*^*+/+*^ and *FUS*^*−/−*^ cells, and only a handful of these were rescued by FUS reexpression (Fig. S10, *D*–*F*). While the upregulation of repressed neuronal genes might be expected to occur in late-replicating chromatin, the neuron-related genes in LRD-FUS intervals exhibited both upregulation and downregulation in *FUS*^*−/−*^ cells. These findings may be relevant to understanding FUS-dependent gene regulation in neurons.

## Discussion

FUS DNA repair functions have been deduced from its PARP-dependent recruitment to sites of microirradiation; its co-IP with repair proteins; and the modest chromosome instability and DSB repair defects of FUS-deficient cells (21, 26, 27, 28, 30, 31, 32, 62). Despite these results, a unifying role for FUS in genome protection has yet to emerge. Using *FUS*^*−/−*^ cells with and without reconstitution, we found that, while FUS may play a supporting role in DSB repair, it is more prominently required for timely DNA replication, which plausibly contributes to genome instability and DDR-related phenotypes ascribed to FUS-deficient cells.

FUS is among the first factors recruited to sites of microirradiation, which is driven through association of FUS RGG domains with PAR chains (26, 27, 28, 30, 63). FUS is also reportedly required for the assembly of IR-induced 53BP1 foci (27), despite the fact that FUS does not accumulate at these structures (26). Results in Fig. S1*A* clearly show that FUS is not required for 53BP1 focus formation. In fact, 53BP1 foci recruitment was more persistent in *FUS*^*−/−*^ cells relative to *FUS*^*+/+*^ controls. This result appears to be congruent with the findings of Altmeyer *et al.* (63) who reported that overexpression of the EWSR LCD suppressed IR-induced 53BP1 focus formation. It is conceivable that FUS inhibits local assembly of 53BP1 complexes and/or limits their lateral spread along damaged chromatin. By contrast, *FUS*^*−/−*^ cells exhibited reduced recruitment of BRCA1 (Fig. S2), which mediates HDR and mutually antagonistic to the 53BP1-RIF1 pathway (64). Despite these modest molecular defects, FUS deficiency does not confer sensitivity to mechanistically distinct genotoxins, including IR, camptothecin, MMC, or CLM (Figs. S3 and S4). Our findings are at odds with a recent report by Levone *et al.* (65) that *FUS*^*−/−*^ HeLa cells are sensitive to DSB-inducing agents and that FUS is required for the recruitment of 53BP1, Ku80, and other DSB repair factors to DNA damage. The use of p53-wildtype (U-2 OS) *versus* p53-inactivated (HeLa) cells or different cell survival assays might underlie differences in genotoxin sensitivity between the studies, whereas discrepant 53BP1 recruitment findings may be related to the use of microirradiation, which induces supraphysiological levels of DSBs and SSBs (66).

**Figure 10:**
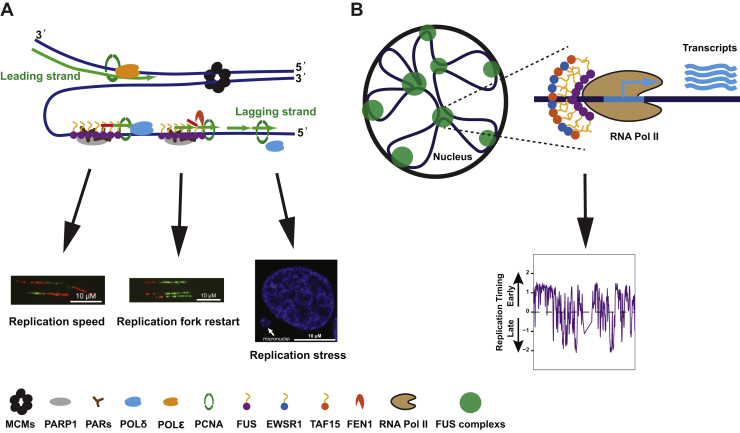
**Working model of FUS in replication progression and replication timing.***A*, based on FUS chromatin proteomics, FUS specifically interacts with POLδ but not POLε. Many replication-coupled single-strand break (SSB) repair factors (PCNA, FEN1, and PARP1) were also enriched with FUS on chromatin. From this, we speculate that FUS facilitates Okazaki fragment processing and PARP-dependent repair of single-strand gaps on the lagging strand (67). Defects in this pathway may contribute to reduced RF speed, RF restart defects, and micronucleus formation. *B*, speculative model for FUS-dependent RT. FUS undergoes phase separation where it may interact transiently recruits RNA polymerase II, potentially in cooperation with EWSR1 and TAF15. Phase-separated FUS complexes (shown in *green circles*) organize chromatin into topologically distinct domains (ERD, MRD, and LRD) that are replicated during early, mid, and late S-phase, respectively. The DNA fiber and micronuclei images were reused from Figure 3 for illustration purpose only. ERD, early replication domain; FEN1, flap endonuclease-1; FUS, fused in sarcoma; LRD, late replication domain; MRD, mid replication domain; PARP1, poly(ADP)-ribosyl (PAR) polymerase 1; PCNA, proliferating cell nuclear antigen; POLδ, polymerase δ; RF, replication fork.

*FUS*^*−/−*^ cells exhibited reduced proliferative potential characterized by reduced RF speed (Fig. 3*A*), delayed RF restart (Fig. 3*B*), reduced expression of S-phase–associated genes (Fig. 4*C*), and reduced loading of pre-RC complexes (Fig. 5*B*). Participation of FUS in DNA replication was suggested by the presence of DNA replication factors in FUS–chromatin complexes (Fig. 6). The association of FUS with lagging strand synthesis factors POLδ1, PCNA, and FEN1, but not leading strand POLε, further suggested that FUS may play a role in the deposition or removal of RNA primers and/or the ligation of single-strand nicks on the lagging strand. It is worth noting that PARP, which was also present in FUS–chromatin complexes, contributes to the ligation of Okazaki fragments on the lagging strand (67). It is also conceivable that FUS plays a role in the postreplication repair of stalled RFs given established roles for POLδ in this process (68). A speculative model depicting a role for FUS in lagging strand synthesis is presented in Figure 10*A*. Alternatively, because FUS did not stably associate with translocating replisomes in the iPOND assay (Fig. S8), it is possible that FUS impacts DNA replication by influencing local chromatin structure or through splicing regulation. Indeed, we identified a handful of DNA replication/repair genes whose alternative splicing was altered in FUS deficiency (Fig. S6, *F*–*Q*). For instance, the inclusion of a poison cassette exon in ORC3 is upregulated in *FUS*^*−/−*^ cells relative to *FUS*^*+/+*^ cells (Fig. S6*K*). Establishing a causal role for splicing changes in the growth and repair defects of FUS-deficient cells awaits future study.

To our knowledge, this is the first study to implicate FUS in the control of RT, a stable cellular characteristic that is established in early G_1_ phase (48, 52, 69). Spatiotemporal control of RT is highly dependent on the master timing factor RIF1, a chromatin-bound factor that also plays important roles in RF stabilization and DSB repair pathway choice (64, 70, 71, 72, 73, 74). RIF1-deficient mammalian cells or yeast exhibit spatial changes in DNA replication that correlate with premature replication origin firing (53, 54, 75, 76, 77). RIF1 is thought to control RT by organizing chromatin domains with shared timing characteristics (78). Like RIF1-deficient cells, *FUS*^*−/−*^ cells exhibited bidirectional RT changes; however, FUS and RIF1 are unlikely to act through the same pathway since they exhibited different intranuclear localization patterns (not shown) and did not detectably interact on chromatin (Fig. 6*A*). In addition, the fact that some chromosomal domains replicate earlier in *FUS*^*−/−*^ cells relative to *FUS*^*+/+*^ cells suggests that the RT functions of FUS are at least partially independent from its positive contributions to DNA replication initiation.

Two plausible models may underlie participation of FUS in RT, with both models invoking the phase-separation characteristics of FUS as a central mechanistic feature (79). First, FUS may fulfill a chromatin-bundling function (53, 78). In this model, the DNA-binding and dynamic oligomerization properties of FUS promote the assembly of chromatin domains that are replicated with similar timing. This role would be conceptually similar to that proposed for RIF1. Nonexclusively, FUS-dependent RT may be linked to its roles in transcriptional activation. The LCDs of FUS, EWSR1, and TAF15 bind to the CTD of RNA pol II (19, 80, 81, 82) and function as potent transcriptional activators when fused to heterologous DNA-binding domains (15, 83, 84). Indeed, transcriptional deregulation is thought to drive malignant transformation in soft-tissue sarcomas harboring oncogenic fusions of FET genes with site-specific transcription factors, such as CHOP, FLI1, and CREB (4, 85, 86). A reciprocal relationship between RT and transcription is supported by studies showing that RT switches during embryonic development precede transcriptional changes of proximal genes (59, 87, 88) and work showing that transcriptional activation leads to RT advancement (89). Sima *et al.* (49) further demonstrated that *cis*-regulatory elements within an enhancer promoted early RT of the *Dppa2/4* domain in mouse ESCs. While absolute levels of transcription were not significantly different, transcriptionally active genes showed delayed RT in *FUS*^*−/−*^ cells relative to *FUS*^*+/+*^ cells (Fig. 9, *F* and *G*).

The LCD of FUS, in addition to intrinsically disordered regions of transcriptional coactivators, BRD4 and MED1, has been implicated in the assembly of phase-separated transcription “condensates” at gene enhancers (90, 91, 92). We speculate that FUS-dependent clustering of transcription complexes and chromatin looping may specify FUS-dependent RDs that undergo coordinate RT regulation (Fig. 10*B*). Chromatin capture approaches, such as ChIA-PET, will be needed to test this hypothesis.

It has been reported that ALS-associated mutations in FUS disrupt DSB repair (27, 65), SSB repair (31), and resolution of R-loops, which are RNA–DNA hybrids that can be processed into DSBs during transcription (93). While our present experiments did not evaluate the replication or repair functions of FUS^ALS^ mutants, the subtle DSB repair and genotoxin sensitivity phenotypes of *FUS*^*−/−*^ cells makes it unlikely that such mutants drive genome stability through a purely LOF mechanism. On the other hand, FUS aggregation in end-stage ALS could confer LOF repair phenotypes or and/or sequester critical repair factors in the cytoplasm. Along these lines, FUS aggregation and nuclear LOF could impact the proliferative capacity, genome stability, and function of glia and other mitotic cell types in the central nervous system. The extent to which neuronal or glial genome instability would drive disease in ALS-FUS relative to other mechanisms, including translational deregulation, splicing deregulation, nuclear import/export deregulation, and proteostasis defects, remains to be determined.

## Experimental procedures

### Cell culture and gene editing

The U-2 OS, NCI-H460, and HEK293T cell lines were obtained from the American Type Culture Collection. U-2 OS and U-2 OS derivative cell lines were grown in McCoy's medium (Corning; 10-050-CV). HEK293T cells were grown in Dulbecco's modified Eagle's medium (Corning; 10-013-CV). NCI-H460 cells were maintained in RPMI1640 medium (Corning; 10-040-CV). All cell lines were grown in medium with 10% fetal bovine serum (Atlanta Biologicals, Inc) and 1% penicillin/streptomycin (Corning; 30-002-CI) and incubated at 37 °C in 5% CO_2._ For G_1_/S synchronization experiments, cells were treated with 2 mM thymidine for 19 h, released into thymidine-free growth media for 9 h, and then returned to thymidine-containing media for an additional 16 h. The cells were washed three times with PBS and then released into complete media for the indicated periods.

*FUS*^*−/−*^ cells were generated by transient transfection of U-2 OS cells with pX459 vectors (v2, Addgene plasmid no. 62988) (94) expressing single guide RNAs (CGCCAGTCGAGCCATATCCC and AGAGCTCCCAATCGTCTTAC) targeting exon 4 using jetPRIME (Polyplus; 114-07). Twenty-four hours after transfection, cells were selected for 72 h with 1 μg/ml puromycin and then diluted into 96-well plates at an average density of one cell per well, and single clones were isolated and screened for FUS knockout by Western blotting. All clones were sequenced around the targeted sequence, and four clones were selected for further study. We reconstituted *FUS*^*−/−*^ Cl.110 with a FUS CDS cloned into a pQCXIH CMV/TO DEST retroviral vector (Addgene; #17394) vector by gateway cloning. As a negative control, the GUS gene from vector pENTRGUS (Invitrogen) was also cloned into pQCXIH CMV/TO DEST vector. The FUS^ΔLCD^ construct was generated by PCR amplification of the FUS CDS beginning at codon 155 and recombination cloning into pQCXIH CMV/TO DEST as described previously. Retroviral plasmids were packaged with GP2-293 packaging cell line (Clonetech; 631458). Stably transduced cells were selected with 50 μg/ml hygromycin for 1 week, and single clones were isolated, expanded, and tested for FUS expression.

### Lentivirus and retrovirus

The pLKO.1 system was used to package lentiviruses and deliver shRNA. The following shRNA target sequences were designed using the RNAi Consortium online tool (Broad Institute) and were cloned into pLKO.1-TRC (Addgene; #10878), according to the manufacturer's suggestions: EWSR1 5′-TGCATTGACTACCAGATTTAT-3′ and TAF15 5′-TGACATGATCCATAGTGAAAT-3′. The nontargeting shRNA (Addgene; #1864) and FUS sequences have been reported previously (26). For pSUPERIOR system, nontargeting 5′-TTCTCCGAACGTGTCACGT-3′, TAF15 5′-ACAGCGGAGATAGAAGTGG-3′. Lentiviral particles were produced by transient transfection of HEK293T cells with pLKO.1, psPAX2 (Addgene; #12260), and VSV-G (Addgene; #8454) in a ratio of 3:2:1. Retrovirus particles were produced as described previously using GP2-293 system.

### EdU labeling, flow cytometry, microscopy, and DNA fiber analysis

For cell cycle progression experiments, U-2 OS cells were incubated with 20 μM EdU for 30 min before collection and then fixed with ice-cold 70% ethanol. EdU detection was performed using the Click-IT Plus EdU Alexa Fluor 647 Flow Cytometry Assay Kit (Life Technologies; C10634). PI was added to a concentration of 50 μg/ml. Flow cytometry was performed on Thermo Fisher Attune, and data were analyzed and organized using FlowJo software (FlowJo, LLC). For *in situ* EdU and BrdU staining, U-2 OS cells were pulse labeled for 30 min with 20 μM BrdU or EdU and fixed with 4% paraformaldehyde. For BrdU detection, cells were then incubated with 2 M HCl for 30 min and then permeabilized with 0.2% Triton-X100 for 15 min at room temperature, washed, and blocked in 3% bovine serum albumin (BSA). Cells were stained with BrdU primary antibody (Santa Cruz; sc-32323) in 3% BSA and incubated overnight at 4 °C, followed by washing in PBS with 0.02% Tween-20 and incubation with appropriate secondary antibodies in 3% BSA for 1 h at room temperature. EdU was detected by click chemistry and described previously. Samples were mounted in VECTASHIELD mounting medium with 4′,6-diamidino-2-phenylindole (DAPI) (Vector; H-1200) before imaging. For general immunostaining experiments, cells were seeded into 12-well plate with glass coverslip (and transferred to a humidity chamber for immunostaining with appropriate antibodies). Nuclear DNA either stained with 0.5 μg/ml DAPI for 10 min at room temperature and then mounted with mounting medium for fluorescence (Vector; H-1000) or directly mounted in mounting medium with DAPI for fluorescence (Vector; H-1200) before imaging. Images were acquired using a Nikon A1RS Confocal Microscope under a 63× oil immersion objective. Images were organized using Fiji ImageJ software. Proximity ligation assay foci were counted in CellProfiler (version 3.1.5). DNA fibers were prepared and analyzed as described (35). In brief, cells were pulsed with 50 μM 5-iodo-2′-deoxyuridine and CldU for times indicated in each experiment. Cells were lyzed directly on glass slides, fixed, denatured, stained, and imaged with Keyence BZ-X710 microscope. Image analysis was done with ImageJ. A minimum of 150 fibers were measured for each independent experiment, and analysis shows mean of three independent experiments (biological replicates).

### RNA-Seq and gene expression

Total RNA was isolated using the TRIzol reagent (Invitrogen; 15596018) following the manufacturer's protocol and treated with TURBO DNase (Invitrogen; AM2239). Then RNA samples were sent to Novogene (Novogene Co, Ltd) for nonstranded cDNA library building and sequencing at PE150 with NovoSeq 6000. Raw read adapters were trimmed by fastp (95) and then were mapped to human genome (GRCh38) by STAR. The number of RNA-Seq reads mapped to each transcript was summarized with featureCounts (96), and differential expression was called using DESeq2 (97). Three biological replicates were used for each sample. Splicing events were identified by MAJIQ2 (98)and filtered with an absolute dPSI ≥20%. GO analysis was performed on MetaScape Web site (99). Signal tracks were visualized by trackViewer (100). For qPCR analysis, total RNA was reverse transcribed into cDNA using SuperScript IV VILO Master Mix with ezDnase enzyme Kit (Invitrogen; 11766050). The primers were designed by Beacon Designer or National Center for Biotechnology Information primer-blast online tool. qPCR reaction was performed on Bio-Rad CFX RealTime PCR system using iTaq Universal SYBR Green Supermix (Bio-Rad; 1725125).

### RT analysis

Cells were prepared and collected accordingly (55) with the following modifications. Approximately 10 million asynchronous cells were collected and fixed in 70% ethanol, washed with ice-cold PBS, and treated with Accutase (CORNING; 25-058-CI) for 20 min at room temperature. Cells were pelleted and resuspended in 2 ml PBS with 250 μl 10 mg/ml RNaseA and incubated at 37 °C for 30 min and stained with PI and then sorted to G_1_- and S-phase fractions by flow cytometry. DNA extracts from sorted cells were prepared using with DNeasy Blood and Tissue Kit (Qiagen; 69504) and single-end 100-base sequencing libraries prepared using TruSeq kit (Illumina), and deep sequencing was performed on HighSeq 2500. The analysis was carried out according to Marchal *et al.* (101). Briefly, reads were trimmed by fastp and mapped onto the human genome (GRCh38) using bowtie2. The RT (S/G_1_ ratio) was calculated in a fixed window size of 20 kb. Then RT raw data were used for quantile normalization and then smoothened with Loess smoothing. The RT signal and replication signal enrichment analysis were performed by deeptools (102). Two biological replicates were analyzed separately. RT domains were identified by unsupervised Segway deep learning tool (56, 57, 58) to *de novo* segment RDs in our samples with the setting: resolution = 1000, num-labels = 3. The running script can be found on GitHub (https://github.com/biofisherman/FusReplication).

### Immunoblotting

For whole-cell extraction, cells were resuspended in high salt lysis buffer (50 mM Tris, pH 7.5, 300 mM NaCl, 10% glycerol, 0.5% Triton X-100, 2 mM MgCl_2_, 3 mM EDTA, 1% Protease Inhibitor Cocktail [Sigma, P8340-5 ml]) supplemented with benzonase (50 U/ml) and incubated on ice for 20 min followed by the addition of 4× SDS-loading buffer and heating at 95 °C for 15 min. For CF, cells were resuspended in cytoskeleton (CSK) buffer (20 mM Hepes–KOH [pH 7.4], 100 mM NaCl, 3 mM MgCl_2_, 300 mM sucrose, and 1% Protease Inhibitor Cocktail [Sigma; P8340-5 ml]) containing 0.5% Triton X-100, incubated on ice for 20 min, and centrifuged for 5 min at 5000*g* at 4 °C. The supernatant was transferred to a new tube and saved as soluble fraction, whereas the pellet/CF was washed twice in CSK buffer without detergent and resuspended in CSK buffer with benzonase (50 U/ml) for 20 min digestion at which time 4× SDS loading buffer was added and the lysates heated to 95 °C for 15 min. For immunoblotting, samples were separated by SDS-PAGE and transferred to polyvinylidene fluoride membranes and immunoblotted with primary antibodies and LI-COR IRDye secondary antibodies (IRDye 800CW goat anti-rabbit and IRDye 680RD goat antimouse) as described (103, 104). Signals were acquired using Odyssey bio-systems (LI-COR Biosciences). Immunoblotting results were analyzed and organized with ImageStudio Lite software (LI-COR Biosciences).

### FUS purification and MS

Rapid IP MS of endogenous proteins assay of FUS was carried out as described (42) with the following modifications. Briefly, ∼20 million cells were counted and fixed with 20 ml 1% formaldehyde solution for 8 min at room temperature. Fixation was quenched by adding 0.12 M glycine. The soluble fraction was extracted in 10 ml of LB1 (50 mM Hepes–KOH [pH 7.5], 140 mM NaCl, 1 mM EDTA, 10% glycerol, 0.5% NP-40, 0.25% Triton X-100, 1% Protease Inhibitor Cocktail [Sigma; P8340-5 ml]) for 10 min with rotation at 4 °C. Cell nuclei were pelleted and washed once with 10 ml LB2 (10 mM Tris–HCl [pH 8.0], 100 mM NaCl, 1 mM EDTA, 0.5 mM EGTA, and 1% Protease Inhibitor Cocktail) and then resuspended in 500 μl LB3 (10 mM Tris–HCl [pH 8.0], 100 mM NaCl, 2.5 mM MgCl_2_, 0.1% [w/v] sodium deoxycholate, 0.5% Triton X-100, and 1% Protease Inhibitor Cocktail) with 500 U benzonase and incubated at room temperature for 30 min. Benzonase was deactivated with 2 mM EDTA and 1 mM EGTA. To this mixture was added 50 μl 10% Triton X-100, 37.5 μl of 4 M NaCl, and LB3 to bring the total lysate volume of each sample to 1 ml. Digested lysates were briefly sonicated using a 10 s/50 s on/off cycle for three times at 40% power and clarified by centrifugation at 20,000*g* for 10 min at 4 °C, and supernatants were incubated with 10 μg FUS antibody (Bethyl; A300-302A) overnight at 4 °C with rotation. Subsequently, 50 μl of prewashed Dynabeads protein G (Invitrogen; 10003D) was added to the lysates and incubated for additional 4 h at 4 °C. For Western blot, beads were washed sequentially with 1 ml LB3 and 1 ml radioimmunoprecipitation assay (RIPA) buffer (50 mM Hepes–KOH [pH 7.5], 0.5 M LiCl, 1 mM EDTA, 1% NP-40, 0.7% [w/v] sodium deoxycholate, and 1% Protease Inhibitor Cocktail) once and boiled in 100 μl 2× SDS buffer. For MS, beads were washed five times with 1 ml RIPA buffer and twice in 1 ml of cold fresh prepared 100 mM ammonium hydrogen carbonate (AMBIC) solution and processed as described (42).

FUS rapid IP MS of endogenous proteins from *FUS*^*+/+*^ and *FUS*^*−/−*^ cells were subjected to tryptic digestion and orbitrap MS using the filter-aided sample preparation method (105). The tryptic digest solution was desalted/concentrated using an Omix 100 μl (80 μg capacity) C18 tip. The solution was pipetted over the C18 bed five times and rinsed three times with water and 0.1% TFA to desalt. The peptides were eluted from the C18 resin into 150 μl 70% acetonitrile, 0.1% TFA, and lyophilized. The peptides were resuspended in 95:5 H_2_O:acetonitrile, 0.2% formic acid, and analyzed in duplicate as described later. Samples were analyzed in duplicate (two technical replicates for each of the three biological replicates) by HPLC–electrospray ionization–MS/MS using a system consisting of a high-performance liquid chromatograph (nanoAcquity: Waters) connected to an electrospray ionization Orbitrap mass spectrometer (Q Exactive HF; Thermo Fisher Scientific). HPLC separation employed a 100 × 365 μm fused silica capillary microcolumn packed with 20 cm of 1.7 μm diameter, 130 Å pore size, C18 beads (Waters BEH), with an emitter tip pulled to approximately 1 μm using a laser puller (Sutter Instruments). Peptides were loaded on column at a flow rate of 400 nl/min for 30 min and then eluted over 120 min at a flow rate of 300 nl/min with a gradient of 5% to 35% acetonitrile, in 0.1% formic acid. Full-mass profile scans were performed in the FT orbitrap between 375 and 1500 *m/z* at a resolution of 120,000, followed by MS/MS HCD scans of the ten highest intensity parent ions at 30% relative collision energy and 15,000 resolution, with a mass range starting at 100 *m/z*. Dynamic exclusion was enabled with a repeat count of one over a duration of 30 s.

The data analysis was performed using MetaMorpheus, version 0.0.303 (106, 107). Peaks were read from the raw files, using ThermoRawFileReader for MS1 peak centroiding. The following search settings were used: protease = trypsin; maximum missed cleavages = 2; minimum peptide length = 7; maximum peptide length = unspecified; initiator methionine behavior = Variable; fixed modifications = Carbamidomethyl on C, Carbamidomethyl on U; variable modifications = Oxidation on M; max mods per peptide = 2; max modification isoforms = 1024; precursor mass tolerance = ±5 PPM; product mass tolerance = ±20 PPM. The search database (canonical human UniProt database downloaded 07/09/2017, appended with common Repository of Adventitious Proteins contaminants) contained 20,336 protein entries. Target peptides below 0.01 peptide spectrum match *q* value were quantified by label-free MS1 peak height with FlashLFQ (108, 109) (included in MetaMorpheus), where the *q* value was estimated from target-decoy competition with sequence-reversed decoys. The mean of two technical replicates of each biological replicate was used, and there were total three biological replicates for following analysis. The proteins with average PSMs in three biological replicates lower than 5 were filtered out for data analysis.

### Cell proliferation and survival assays

For cell proliferation assay, 500 cells were plated in each well of 96-well plate, and each sample had six replicates and monitored for 6 days from day 0 to day 5 by CellTiter-Glo 2.0 Assay (Promega; G9242) according to the manufacturer's instructions. The luminescence was recorded by SpectraMax i3 (Molecular Devices). For cell viability assay, 1000 cells/well were plated in 96-well plate with drug-free medium, and varying amounts of drugs were added after 12 h in fresh medium. Cell survival was assayed as same as cell proliferation assay after 3 or 5 days as indicated in the legend to the figure. Data were analyzed and organized by Prism 8.

### iPOND assay

The iPOND experiments were performed as described (35, 110) with minor modifications. Briefly, ∼10^8^ HEK293T cells were pulse labeled with 20 μM EdU for 15 min followed by a 1 h chase with 20 μM thymidine. To induce replication stress, cells were treated with 2 mM HU after EdU labeling for 2 h, and then chased with 20 μM thymidine for 1 h. Each plate was crosslinked with 10 ml 1% formaldehyde in PBS for 20 min and quenched by adding 1 ml of 1.25 M glycine for 5 min. The conjugation of biotin to EdU was carried out by click chemistry reaction for 2 h at room temperature in click reaction buffer (10 μM biotin–azide, 10 mM sodium-l-ascorbate, 2 mM CuSO_4_, and 800 μM Tris(3-hydroxypropyltriazolylmethyl)amine in PBS) and followed by washing once in 0.5% BSA in PBS and once in PBS. Cells were resuspended in LB3 with 500 U benzonase (Santa Cruz; sc-202391) and incubated at room temperature for 30 min. Digested lysates were briefly sonicated using a 10 s/50 s on/off cycle for four times at 40% power and clarified by centrifugation at 8000*g* for 10 min at 4 °C, and supernatants were incubated overnight with 50 μl magnetic streptavidin beads (Dynabeads MyOne Streptavidin T1; 65601) at 4 °C with rotating. Beads were washed once in 1 ml washing buffer (20 mM Tris–HCl [pH 8.0], 500 mM NaCl, 2 mM EDTA, 0.1% [w/v] sodium deoxycholate, and 1% Triton X-100), once with 1 ml RIPA buffer (50 mM Hepes–KOH [pH 7.5], 0.5 M LiCl, 1 mM EDTA, 1% NP-40, 0.7% [w/v] sodium deoxycholate, and 1% Protease Inhibitor Cocktail) and twice in LB3 buffer. Proteins were eluted by boiling in 2× SDS buffer for 25 min.

### Statistical processing

Statistical analysis information including individual replicates and biological replicates number, mean or median, and error bars are explained in the legends to the figures. The statistical tests and resulting *p* values are shown in the legends to the figures and/or figure panels.

## Data availability

All the source data represented in the figures and bioinformatics analysis scripts are available on GitHub (https://github.com/biofisherman/FusReplication). Accession numbers: RT sequencing data have been deposited in the National Center for Biotechnology Information under accession code PRJNA615974. RNA-Seq data accession code is GSE147784 in Gene Expression Omnibus. The MS raw data have been deposited to MassIVE, and the access ID is MSV000087698.

## Supporting information

This article contains supporting information (56, 96, 97, 98, 100, 102, 111, 112, 113, 114, 115, 116).

## Conflict of interest

The authors declare that they have no conflicts of interest with the contents of this article.
