# Supplementary material for: Fused in sarcoma regulates DNA replication timing and kinetics
Source: J Biol Chem. 2021 Aug 8;297(3):101049. doi: 10.1016/j.jbc.2021.101049 (PMC8403768; doi:10.1016/j.jbc.2021.101049)
Supplement: Supplemental Figures S1–S10 [file mmc1.pdf]

Supporting Information

Fused in sarcoma (FUS) regulates DNA replication timing and kinetics

Weiyan Jia[1], Sang Hwa Kim[1], Mark A. Scalf[2], Peter Tonzi[4], Robert J. Millikin[2], William M. Guns[1], Lu Liu[3], Adam S. Mastrocola[1], Lloyd M. Smith[2], Tony T. Huang[4] and Randal S. Tibbetts[1*]

Figure S1

A

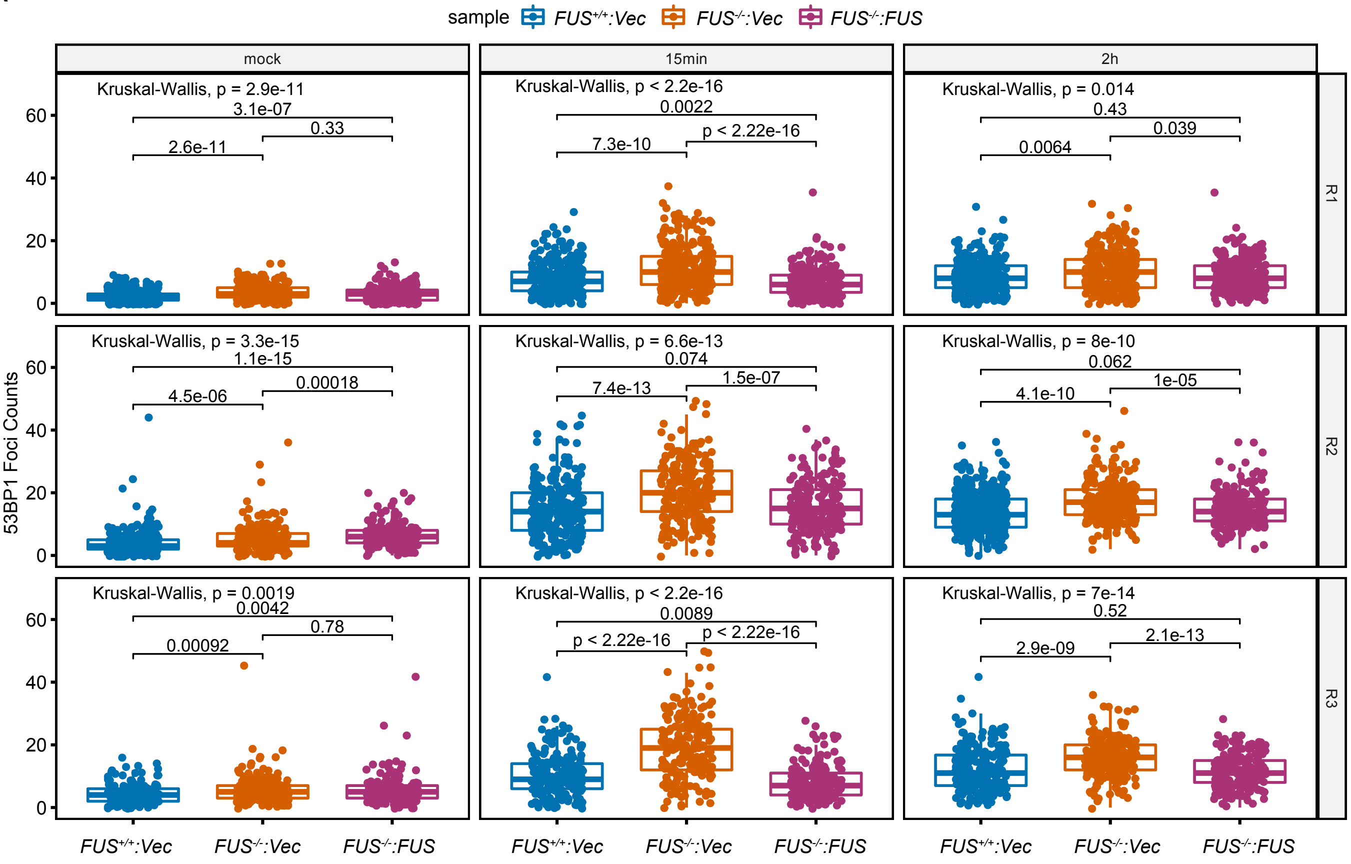
sample FUS+/+:Vec FUS-/-:Vec FUS-/-:FUS
mock
15min
2h
R1
R2
R3
53BP1 Foci Counts
Kruskal-Wallis, p = 2.9e-11
3.1e-07
0.33
2.6e-11
Kruskal-Wallis, p < 2.2e-16
0.0022
p < 2.22e-16
7.3e-10
Kruskal-Wallis, p = 0.014
0.43
0.039
0.0064
Kruskal-Wallis, p = 3.3e-15
1.1e-15
0.00018
4.5e-06
Kruskal-Wallis, p = 6.6e-13
0.074
1.5e-07
7.4e-13
Kruskal-Wallis, p = 8e-10
0.062
1e-05
4.1e-10
Kruskal-Wallis, p = 0.0019
0.0042
0.78
0.00092
Kruskal-Wallis, p < 2.2e-16
0.0089
p < 2.22e-16
p < 2.22e-16
Kruskal-Wallis, p = 7e-14
0.52
2.1e-13
2.9e-09
0
20
40
60
FUS+/+:Vec
FUS-/-:Vec
FUS-/-:FUS

B

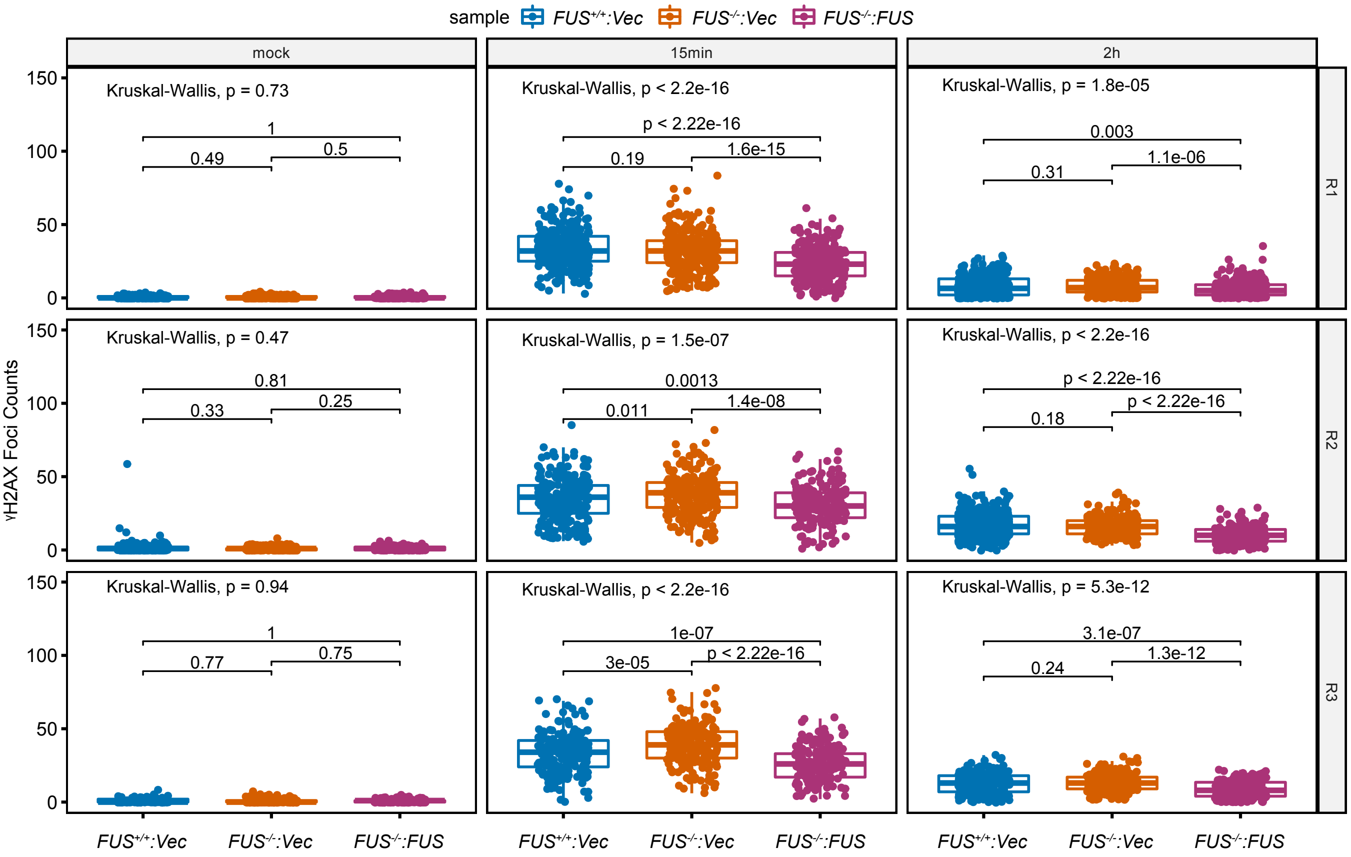
sample FUS+/+:Vec FUS-/-:Vec FUS-/-:FUS
mock
15min
2h
R1
R2
R3
γH2AX Foci Counts
Kruskal-Wallis, p = 0.73
1
0.49
0.5
Kruskal-Wallis, p < 2.2e-16
p < 2.22e-16
0.19
1.6e-15
Kruskal-Wallis, p = 1.8e-05
0.003
0.31
1.1e-06
Kruskal-Wallis, p = 0.47
0.81
0.33
0.25
Kruskal-Wallis, p = 1.5e-07
0.0013
0.011
1.4e-08
Kruskal-Wallis, p < 2.2e-16
p < 2.22e-16
p < 2.22e-16
0.18
Kruskal-Wallis, p = 0.94
1
0.77
0.75
Kruskal-Wallis, p < 2.2e-16
1e-07
3e-05
p < 2.22e-16
Kruskal-Wallis, p = 5.3e-12
3.1e-07
0.24
1.3e-12
0
50
100
150
FUS+/+:Vec
FUS-/-:Vec
FUS-/-:FUS

Figure S1

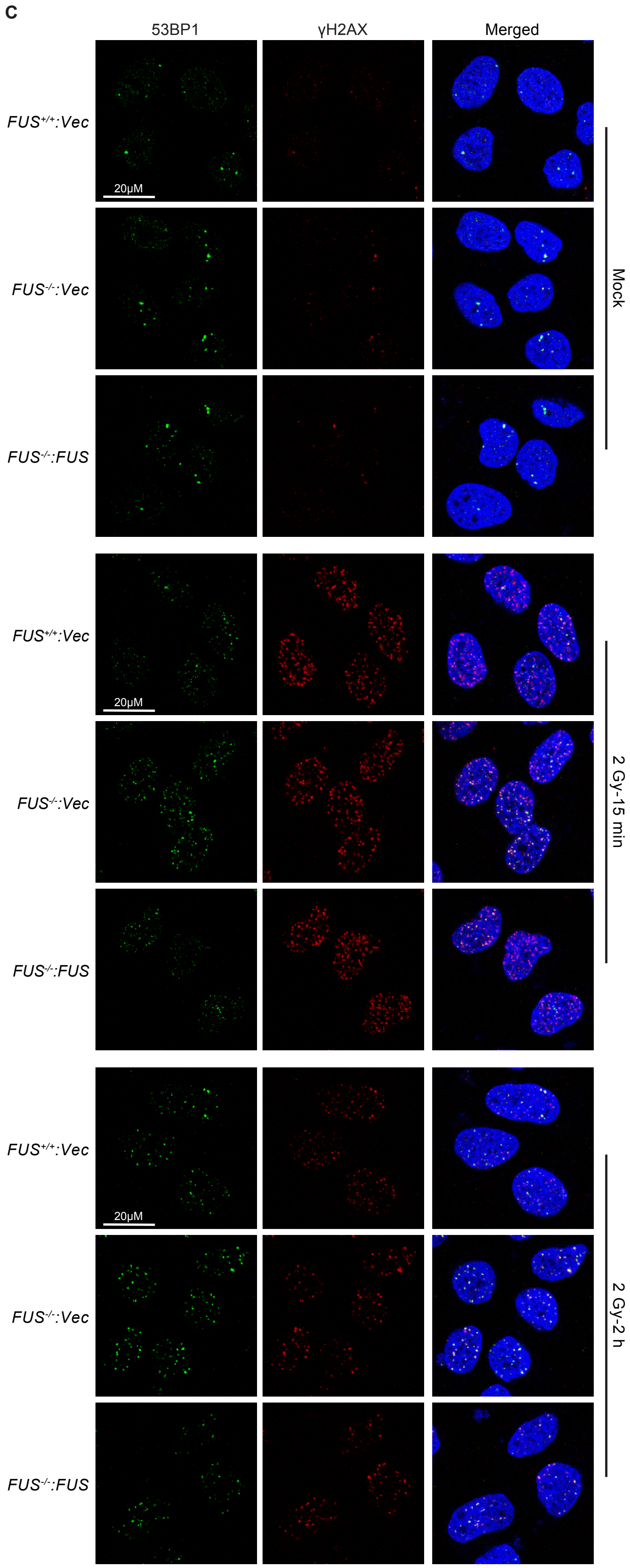
C
53BP1
γH2AX
Merged
FUS+/+:Vec
FUS-/-:Vec
FUS-/-:FUS
FUS+/+:Vec
FUS-/-:Vec
FUS-/-:FUS
FUS+/+:Vec
FUS-/-:Vec
FUS-/-:FUS
20μM
20μM
20μM
Mock
2 Gy-15 min
2 Gy-2 h

Figure S1. Enhanced recruitment and persistence of 53BP1 foci in irradiated *FUS*$^{-/-}$ U-2 OS cells. U-2 OS cells were irradiated with 2 Gy IR, harvested at indicated times, and processed for immunostaining with 53BP1 and γH2AX antibodies. *A,* Quantification of 53BP1 foci. Significance was calculated using Kruskal-Wallis test and Wilcox test was used for comparison between two samples. Bars represent the median. Three biological replicates (R1, R2 and R3) were performed and analyzed separately. *B,* Quantification of γH2AX foci using the same statistical analysis performed in (A). *C,* Representative imaging results for 53BP1 and γH2AX foci data used in panels A and B.

Figure S2

A

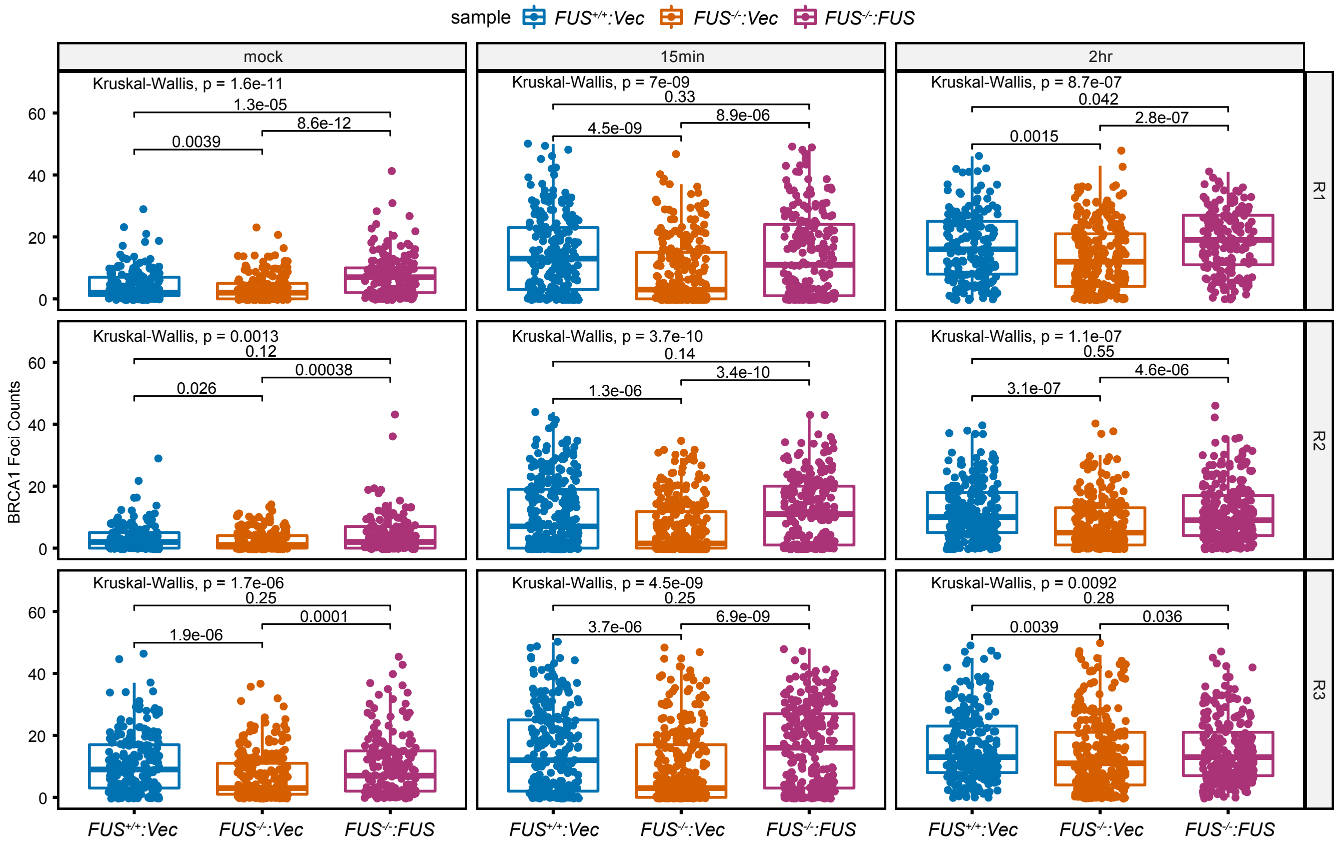
sample FUS+/+:Vec FUS-/-:Vec FUS-/-:FUS
mock
15min
2hr
R1
R2
R3
BRCA1 Foci Counts
Kruskal-Wallis, p = 1.6e-11
Kruskal-Wallis, p = 7e-09
Kruskal-Wallis, p = 8.7e-07
Kruskal-Wallis, p = 0.0013
Kruskal-Wallis, p = 3.7e-10
Kruskal-Wallis, p = 1.1e-07
Kruskal-Wallis, p = 1.7e-06
Kruskal-Wallis, p = 4.5e-09
Kruskal-Wallis, p = 0.0092
FUS+/+:Vec
FUS-/-:Vec
FUS-/-:FUS

B

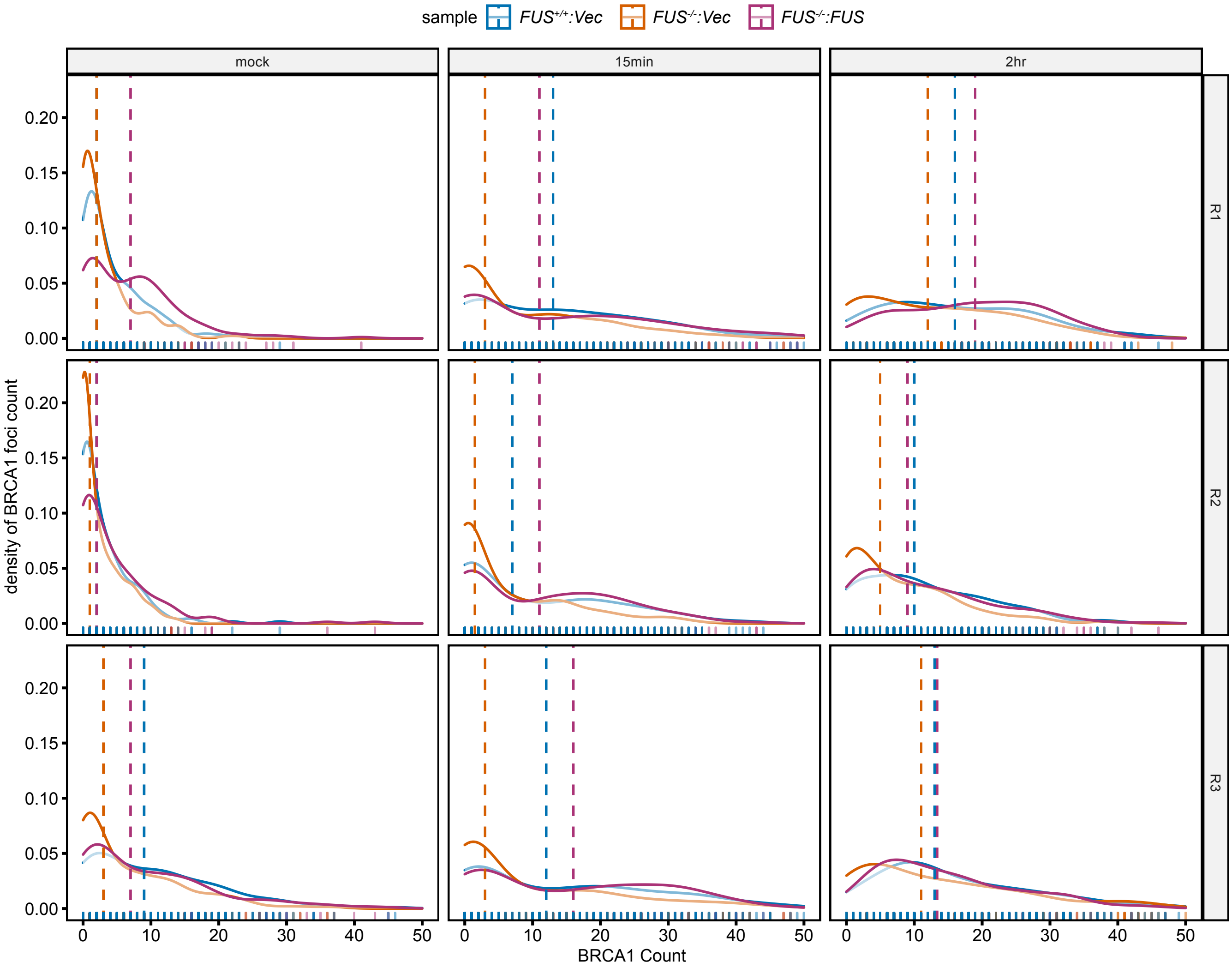
sample FUS+/+:Vec FUS-/-:Vec FUS-/-:FUS
mock
15min
2hr
R1
R2
R3
density of BRCA1 foci count
BRCA1 Count

Figure S2

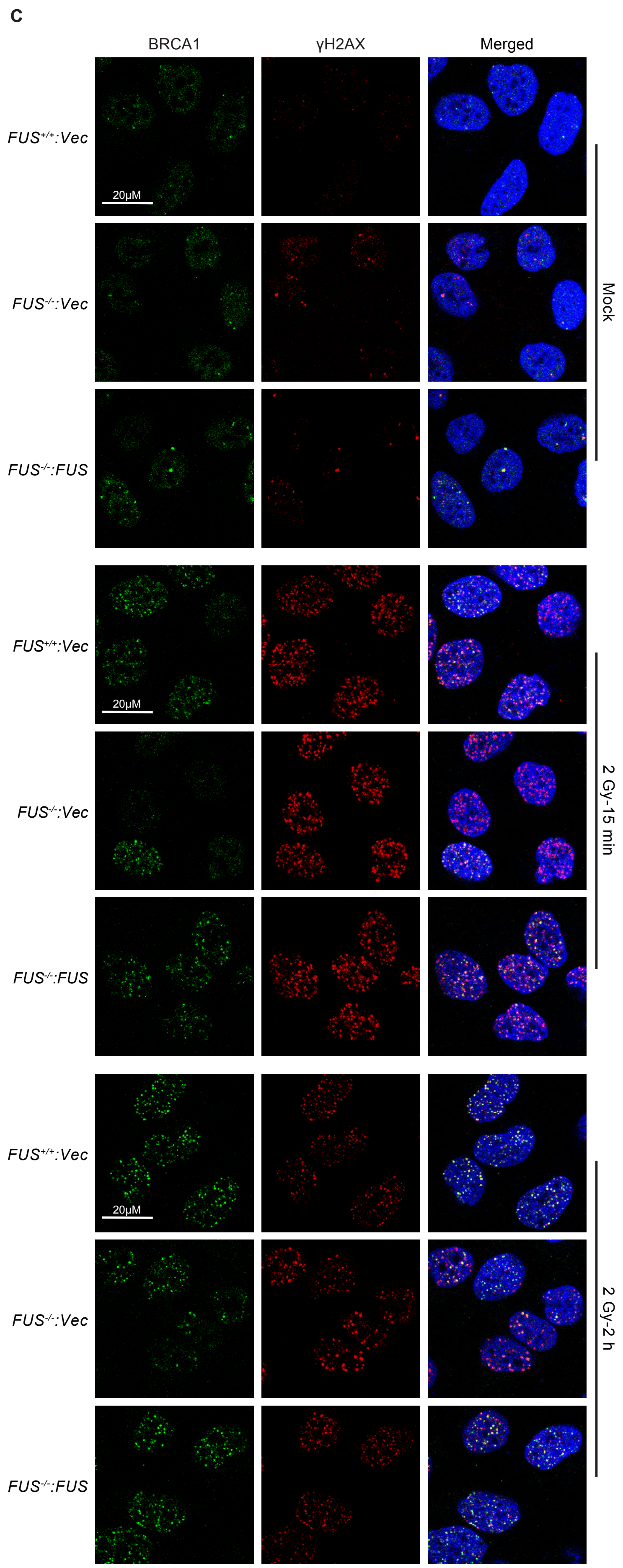

C
BRCA1
γH2AX
Merged
FUS+/+:Vec
FUS-/-:Vec
FUS-/-:FUS
20μM
Mock
2 Gy-15 min
2 Gy-2 h

Figure S2

D

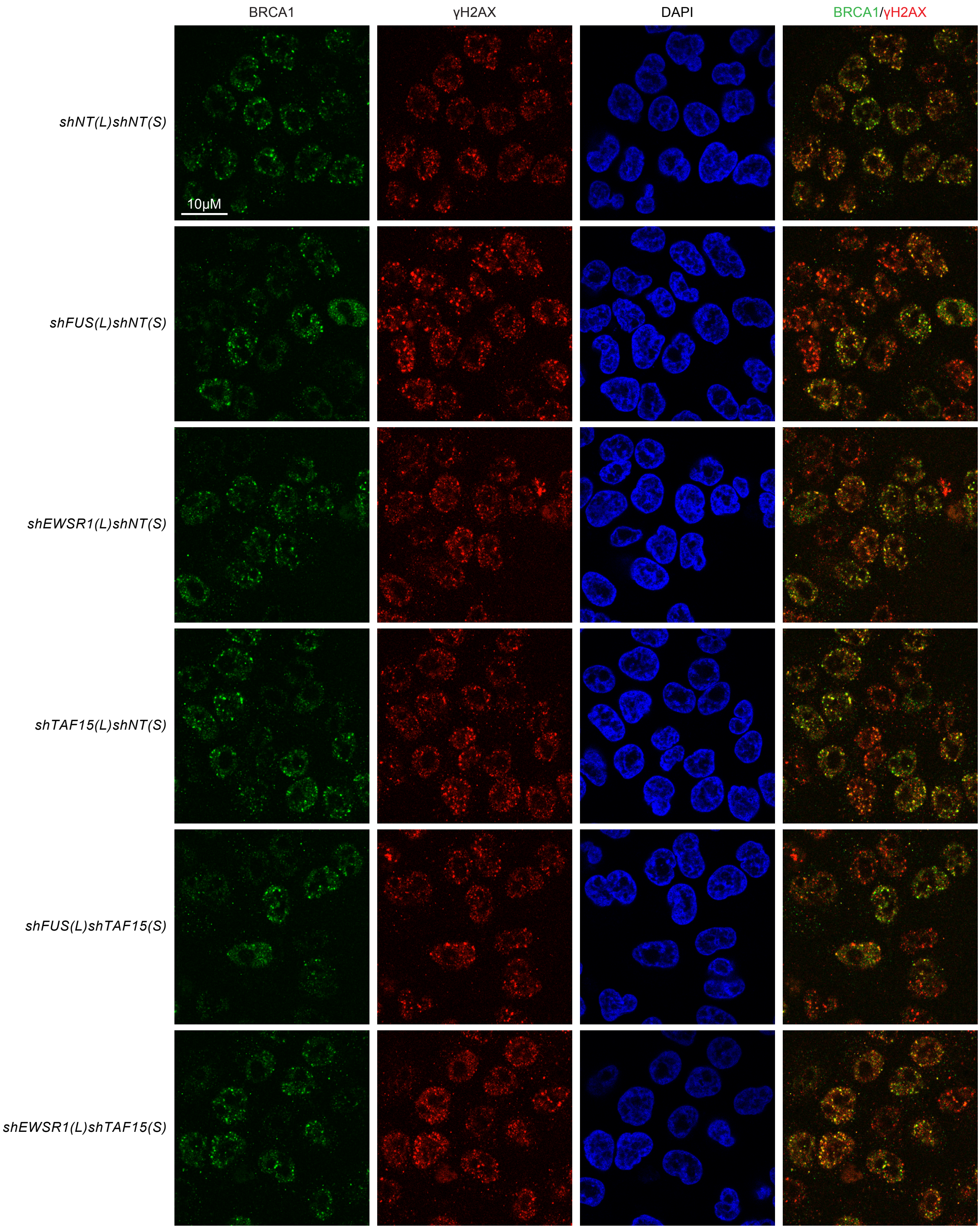
BRCA1
γH2AX
DAPI
BRCA1/γH2AX
shNT(L)shNT(S)
shFUS(L)shNT(S)
shEWSR1(L)shNT(S)
shTAF15(L)shNT(S)
shFUS(L)shTAF15(S)
shEWSR1(L)shTAF15(S)
10μM

Figure S2

E

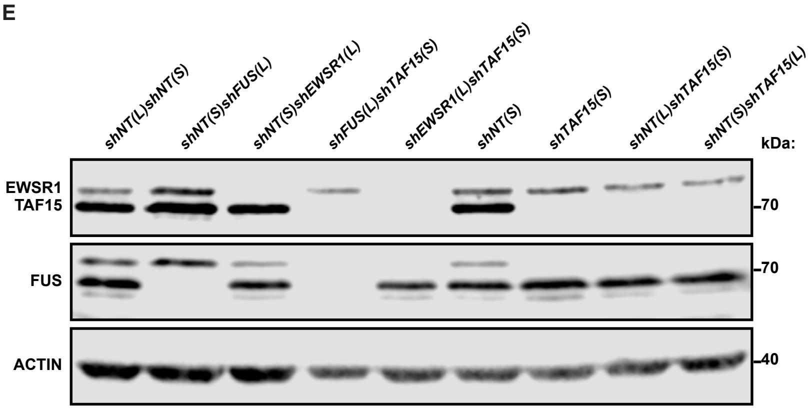
shNT(L)shNT(S)
shNT(S)shFUS(L)
shNT(S)shEWSR1(L)
shFUS(L)shTAF15(S)
shEWSR1(L)shTAF15(S)
shNT(S)
shTAF15(S)
shNT(L)shTAF15(S)
shNT(S)shTAF15(L)
kDa:
EWSR1
TAF15
-70
FUS
-70
ACTIN
-40

F

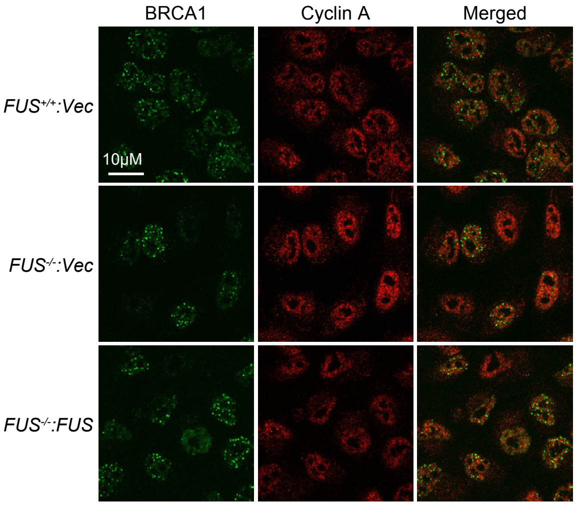
BRCA1
Cyclin A
Merged
FUS+/+:Vec
10μM
FUS-/-:Vec
FUS-/-:FUS

Figure S2. FUS promotes BRCA1 recruitment to DNA damage sites. U-2 OS cells were treated with 2 Gy IR as in Sup. Figure 1 and stained with BRCA1 and γH2AX antibodies. *A,* Quantification of BRCA1 foci. Significance was calculated with Kruskal-Wallis test and a Wilcox test was applied for comparison between two samples. Bars represent the median. Three biological replicates (R1, R2 and R3) were performed and plotted separately. *B,* Density distribution plots of BRCA1 foci counts in each cell. Dashed lines represent the median of BRCA1 foci in each sample. *C,* Representative imaging results for BRCA1 and γH2AX foci *D,* Comparison of BRCA1 focus formation in H460 cells transduced with FUS, EWRS1, and TAF15 shRNA vectors. Cells were treated with 2Gy IR and stained with BRCA1 and γH2AX after recovering for 30 min. NT, non-targeting shRNA; L, pLKO.1-puro vector; S, pSUPERIOR-retro-neo vector. E, Expression levels of FUS, EWSR1, and TAF15 in respective knockdown lines were determined by Western blotting of whole-cell extracts. Actin was used as a loading control. *F*, FUS reexpression reverses the BRCA1 focus formation defect *FUS*$^{-/-}$ cells in of S/G_2-phase. Cells were fixed and stained with BRCA1 and Cyclin A antibodies 15 min after exposure to 2 Gy IR.

Figure S3

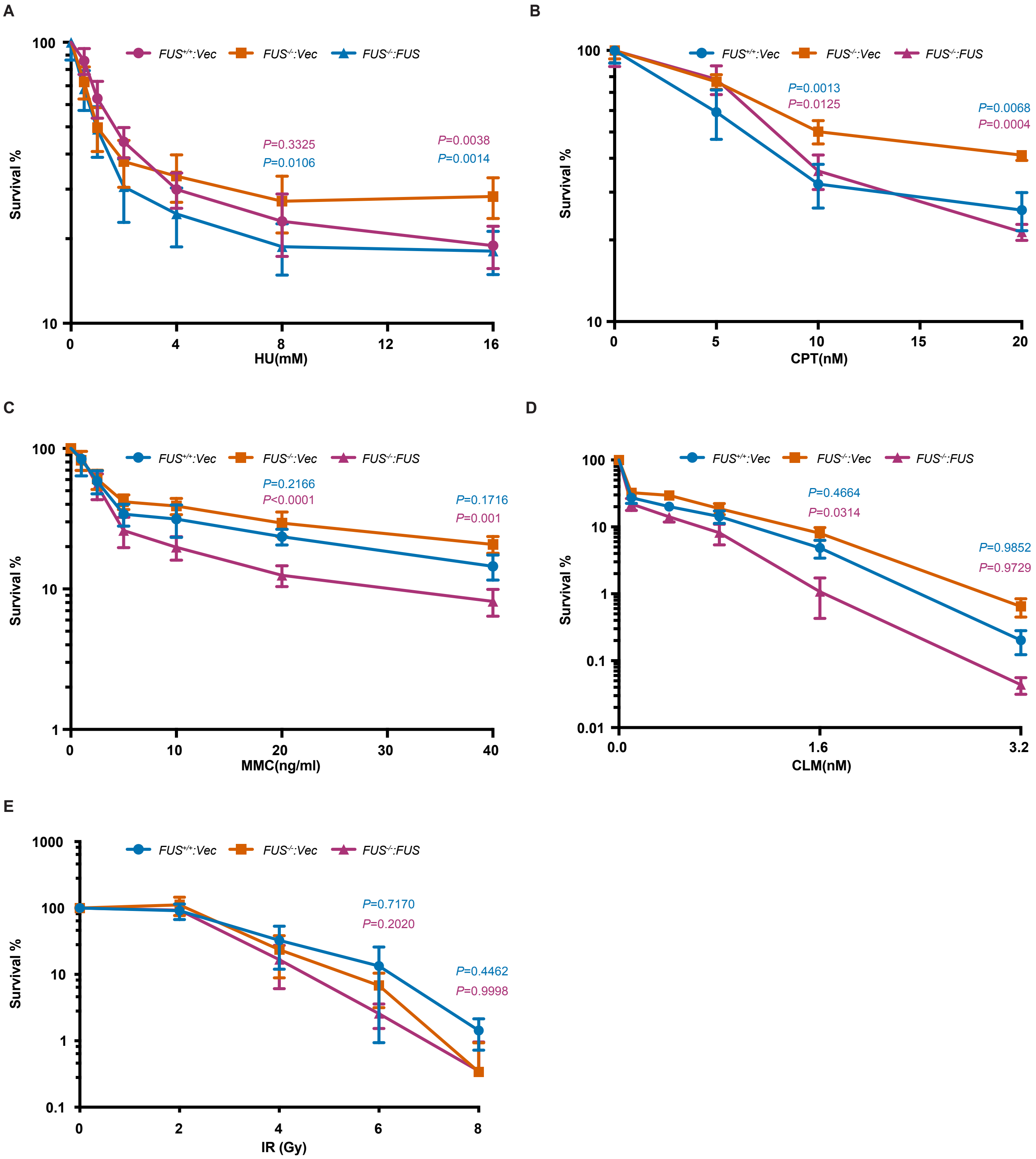
A
FUS+/+:Vec
FUS-/-:Vec
FUS-/-:FUS
Survival %
HU(mM)
P=0.3325
P=0.0106
P=0.0038
P=0.0014
B
CPT(nM)
P=0.0013
P=0.0125
P=0.0068
P=0.0004
C
MMC(ng/ml)
P=0.2166
P<0.0001
P=0.1716
P=0.001
D
CLM(nM)
P=0.4664
P=0.0314
P=0.9852
P=0.9729
E
IR (Gy)
P=0.7170
P=0.2020
P=0.4462
P=0.9998

Figure S3. Sensitivity of *FUS*$^{-/-}$ cells to genotoxins. *A to D,* Cell viability was assessed after 5-days of continuous treatment with HU, CPT, MMC or CLM at the indicated concentration using a CellTiter-Glo assay (Promega). 1000 cells were plated in each well. HU and MMC treatments employed three biological replicates of 5 individual wells each. CPT and CLM employed one biological replicate with 5 individual wells. The bars represent mean ± SD. *E, Clonogenic survival assay under IR treatment. 200, 400, 400, 800 and 800 cells were plated for 0 Gy, 2Gy, 4Gy, 6Gy and 8 Gy condition in 6-well-plates on Day 1. Cells were irradiated on Day 2. Cells were fixed and stained on Day 14.* The Two-way ANOVA test was performed, and *p*-values were adjusted using Tukey's multiple comparisons test. The blue *p*-values compare *FUS*$^{+/+}$*:Vec* and *FUS*$^{-/-}$*:Vec* cells while green *p*-values compared *FUS*$^{-/-}$*:FUS* to *FUS*$^{-/-}$*:Vec* cells.

Figure S4

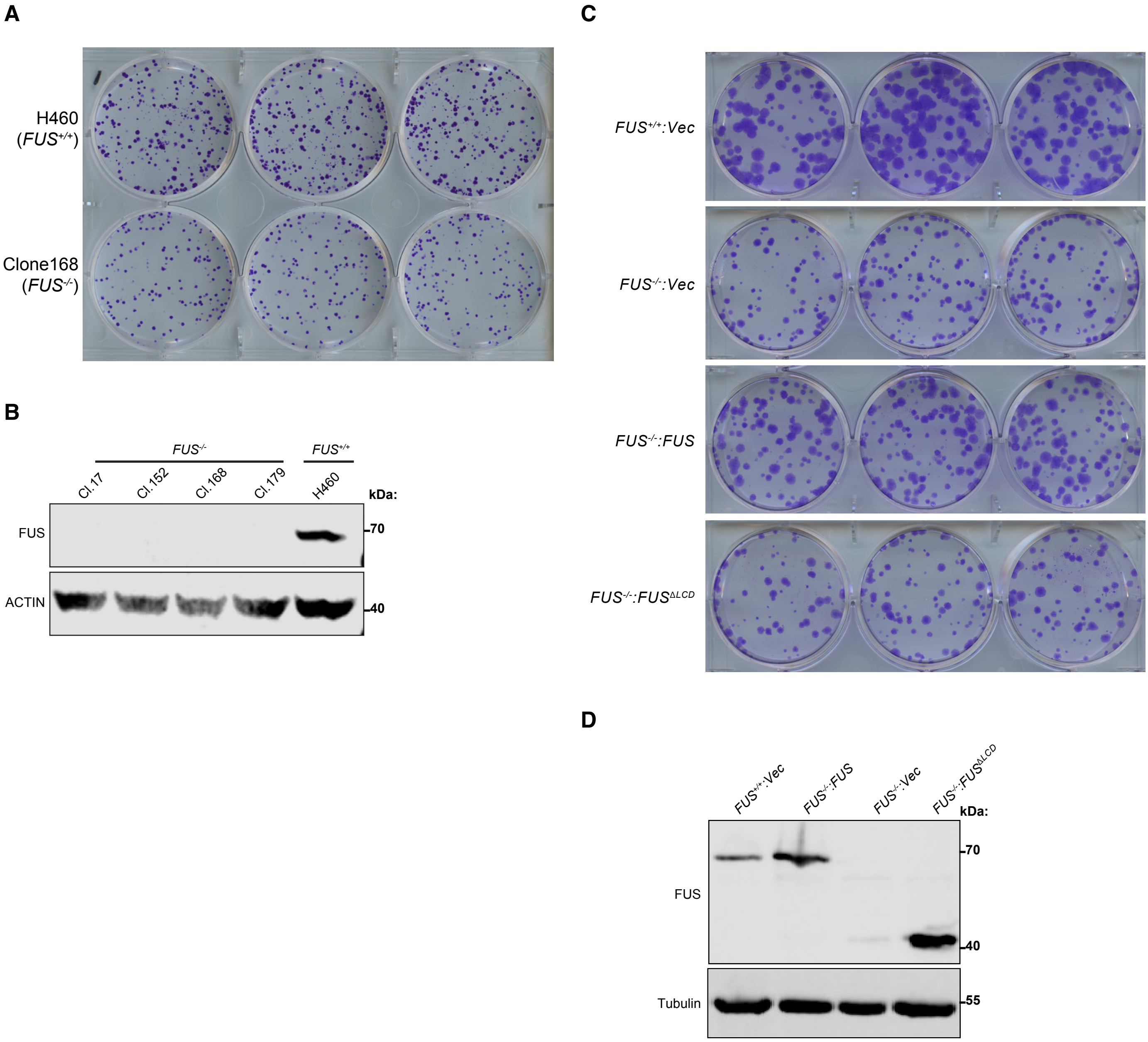
A
H460
(FUS+/+)
Clone168
(FUS-/-)
B
FUS-/-
FUS+/+
Cl.17
Cl.152
Cl.168
Cl.179
H460
kDa:
FUS
70
ACTIN
40
C
FUS+/+:Vec
FUS-/-:Vec
FUS-/-:FUS
FUS-/-:FUSΔLCD
D
FUS+/+:Vec
FUS-/-:FUS
FUS-/-:Vec
FUS-/-:FUSΔLCD
kDa:
FUS
70
40
Tubulin
55

Figure S4. Cell growth Assay. *A,* Colony growth assay of *FUS*$^{+/+}$ and *FUS*$^{-/-}$ (clone 168) H460 cells. *B,* Western blotting results using extracts prepared from independent *FUS*$^{-/-}$ H460 clones and *FUS*$^{+/+}$ H460 cells. *C*, Colony growth assay of *FUS*$^{+/+}$*:Vec*, *FUS*$^{-/-}$*:Vec, FUS*$^{-/-}$*:FUS* and *FUS*$^{-/-}$*:FUS*$^{\Delta PLD}$ cells. *D*, Western blotting of FUS in reconstituted cells used for colony assay in (*C*).

Figure S5

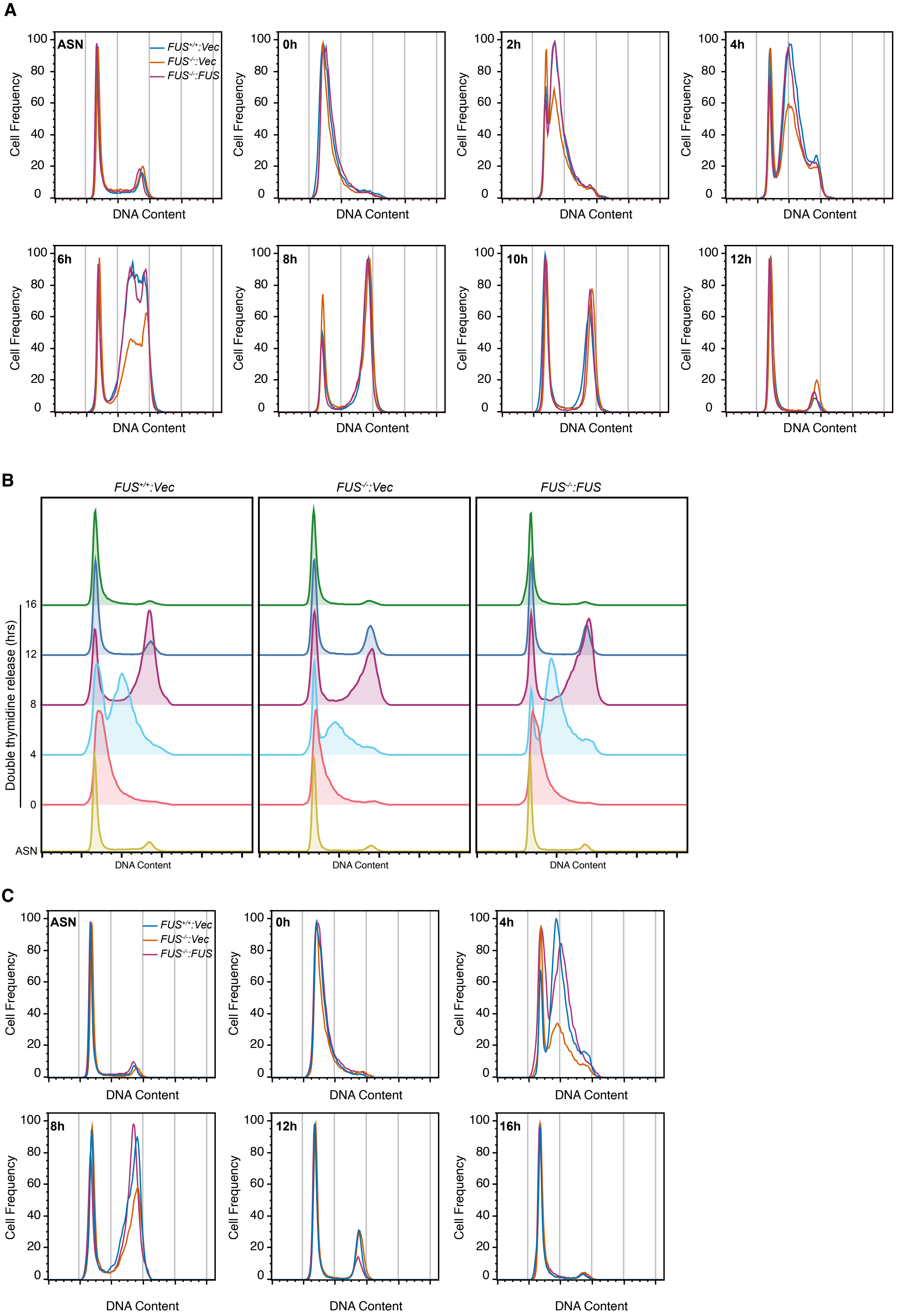
A
ASN
0h
2h
4h
6h
8h
10h
12h
FUS+/+:Vec
FUS-/-:Vec
FUS-/-:FUS
Cell Frequency
DNA Content
0
20
40
60
80
100
B
FUS+/+:Vec
FUS-/-:Vec
FUS-/-:FUS
Double thymidine release (hrs)
ASN
0
4
8
12
16
DNA Content
C
ASN
0h
4h
8h
12h
16h
FUS+/+:Vec
FUS-/-:Vec
FUS-/-:FUS
Cell Frequency
DNA Content

Figure S5

D

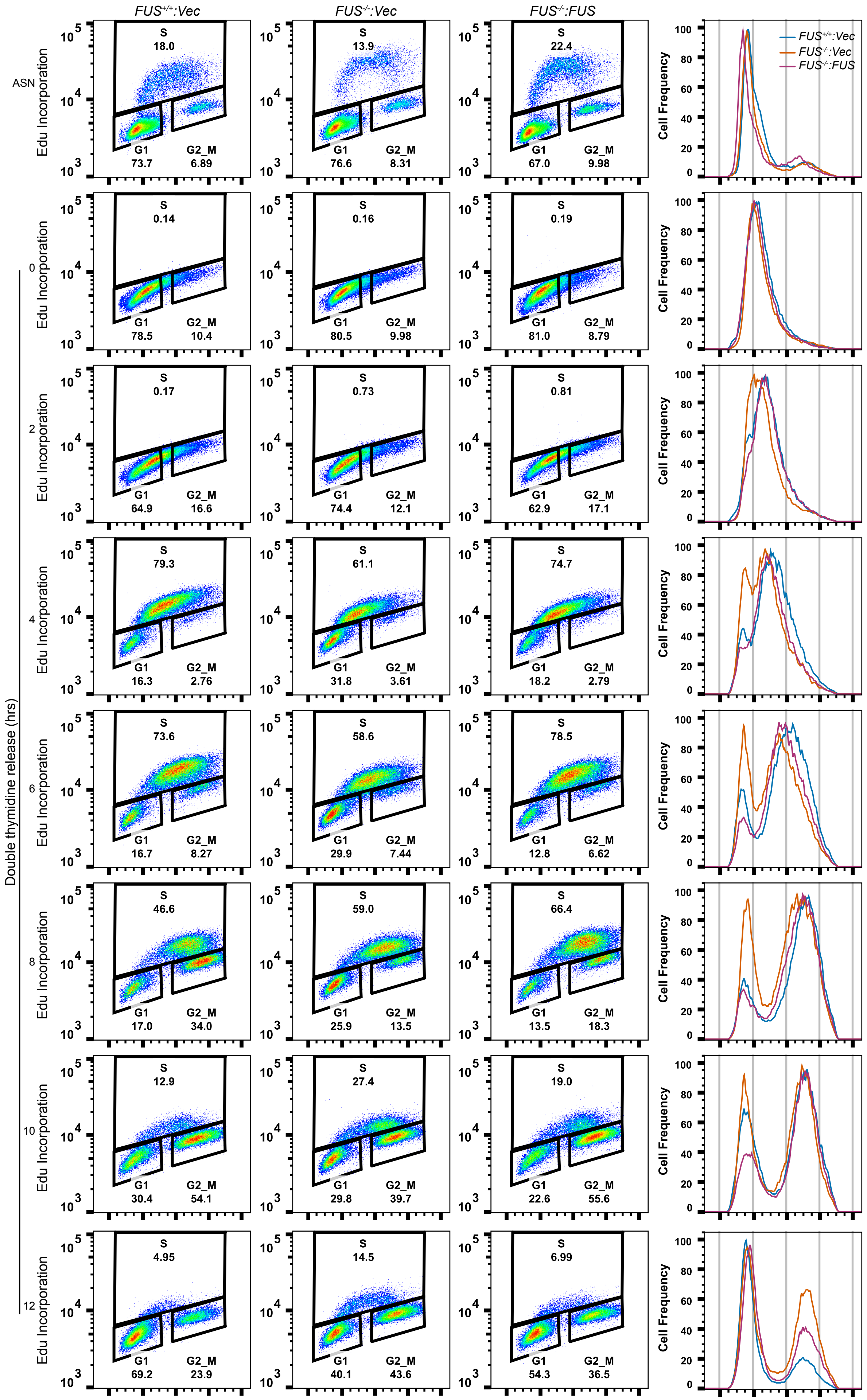

FUS+/+:Vec
FUS-/-:Vec
FUS-/-:FUS
ASN
Double thymidine release (hrs)
0
2
4
6
8
10
12
Edu Incorporation
Cell Frequency
S
G1
G2_M
18.0 73.7 6.89
13.9 76.6 8.31
22.4 67.0 9.98
0.14 78.5 10.4
0.16 80.5 9.98
0.19 81.0 8.79
0.17 64.9 16.6
0.73 74.4 12.1
0.81 62.9 17.1
79.3 16.3 2.76
61.1 31.8 3.61
74.7 18.2 2.79
73.6 16.7 8.27
58.6 29.9 7.44
78.5 12.8 6.62
46.6 17.0 34.0
59.0 25.9 13.5
66.4 13.5 18.3
12.9 30.4 54.1
27.4 29.8 39.7
19.0 22.6 55.6
4.95 69.2 23.9
14.5 40.1 43.6
6.99 54.3 36.5

Figure S5. S phase progression defects in *FUS*$^{-/-}$ U-2 OS cells. *A,* Overlay of cell cycle profiles of *FUS*$^{+/+}$, *FUS*$^{-/-}$, and *FUS*$^{-/-}$*:FUS* cells released from a double thymidine block (See Fig. 2A). *B,* An independent replicate of DNA replication progression results presented in Figure 2A. *C,* Overlay of cell cycle profiles presented in panel B. *D,* Full time course analysis of cell cycle progression profiles between *FUS*$^{+/+}$, *FUS*$^{-/-}$, and *FUS*$^{-/-}$*:FUS* cells that is presented in Figure 2B.

Figure S6

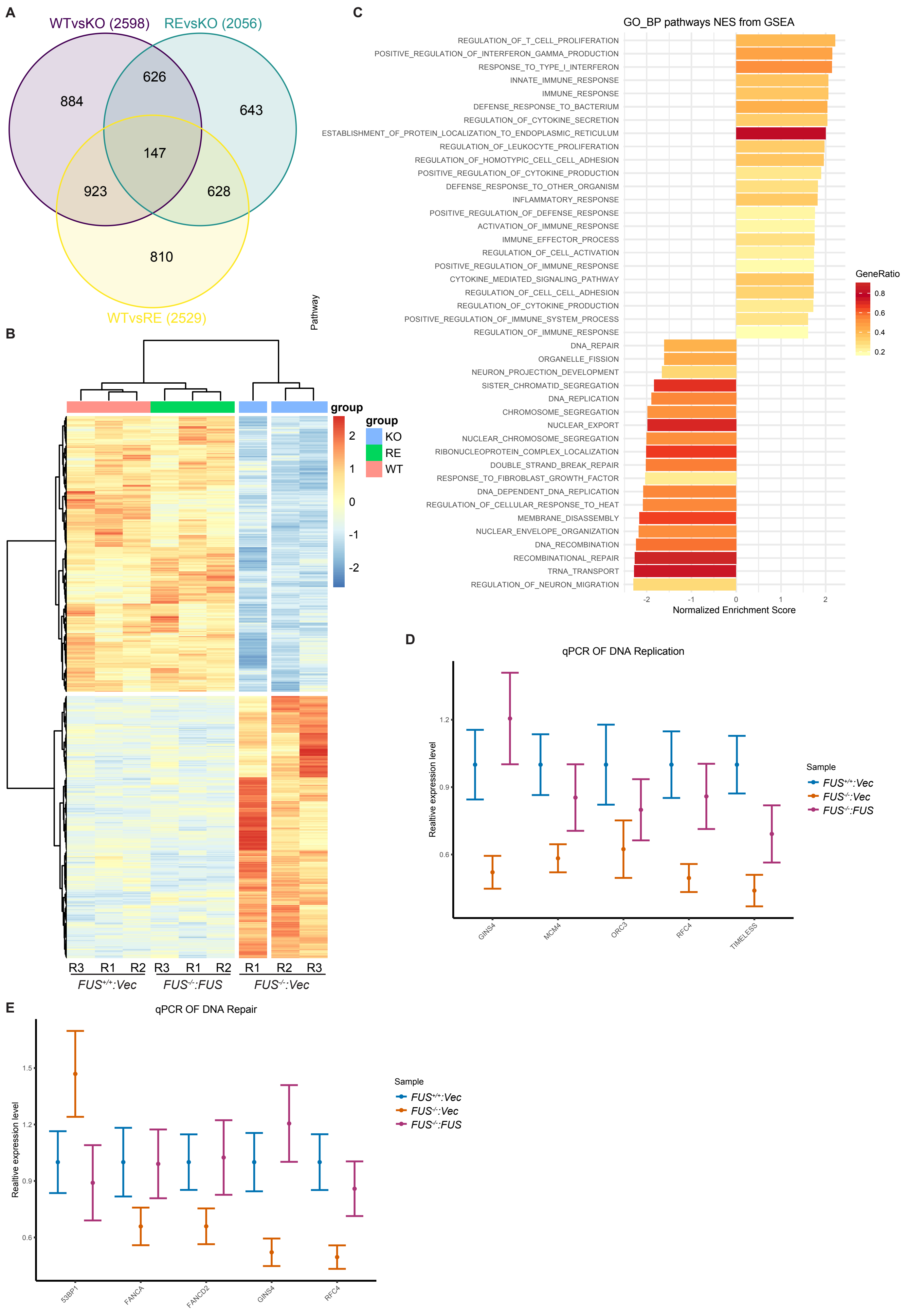
A
WTvsKO (2598)
REvsKO (2056)
WTvsRE (2529)
884
626
643
147
923
628
810
B
group
KO
RE
WT
2
1
0
-1
-2
R3 R1 R2 R3 R1 R2 R1 R2 R3
FUS+/+:Vec
FUS-/-:FUS
FUS-/-:Vec
C
GO_BP pathways NES from GSEA
Pathway
REGULATION_OF_T_CELL_PROLIFERATION
POSITIVE_REGULATION_OF_INTERFERON_GAMMA_PRODUCTION
RESPONSE_TO_TYPE_I_INTERFERON
INNATE_IMMUNE_RESPONSE
IMMUNE_RESPONSE
DEFENSE_RESPONSE_TO_BACTERIUM
REGULATION_OF_CYTOKINE_SECRETION
ESTABLISHMENT_OF_PROTEIN_LOCALIZATION_TO_ENDOPLASMIC_RETICULUM
REGULATION_OF_LEUKOCYTE_PROLIFERATION
REGULATION_OF_HOMOTYPIC_CELL_CELL_ADHESION
POSITIVE_REGULATION_OF_CYTOKINE_PRODUCTION
DEFENSE_RESPONSE_TO_OTHER_ORGANISM
INFLAMMATORY_RESPONSE
POSITIVE_REGULATION_OF_DEFENSE_RESPONSE
ACTIVATION_OF_IMMUNE_RESPONSE
IMMUNE_EFFECTOR_PROCESS
REGULATION_OF_CELL_ACTIVATION
POSITIVE_REGULATION_OF_IMMUNE_RESPONSE
CYTOKINE_MEDIATED_SIGNALING_PATHWAY
REGULATION_OF_CELL_CELL_ADHESION
REGULATION_OF_CYTOKINE_PRODUCTION
POSITIVE_REGULATION_OF_IMMUNE_SYSTEM_PROCESS
REGULATION_OF_IMMUNE_RESPONSE
DNA_REPAIR
ORGANELLE_FISSION
NEURON_PROJECTION_DEVELOPMENT
SISTER_CHROMATID_SEGREGATION
DNA_REPLICATION
CHROMOSOME_SEGREGATION
NUCLEAR_EXPORT
NUCLEAR_CHROMOSOME_SEGREGATION
RIBONUCLEOPROTEIN_COMPLEX_LOCALIZATION
DOUBLE_STRAND_BREAK_REPAIR
RESPONSE_TO_FIBROBLAST_GROWTH_FACTOR
DNA_DEPENDENT_DNA_REPLICATION
REGULATION_OF_CELLULAR_RESPONSE_TO_HEAT
MEMBRANE_DISASSEMBLY
NUCLEAR_ENVELOPE_ORGANIZATION
DNA_RECOMBINATION
RECOMBINATIONAL_REPAIR
TRNA_TRANSPORT
REGULATION_OF_NEURON_MIGRATION
-2 -1 0 1 2
Normalized Enrichment Score
GeneRatio
0.8
0.6
0.4
0.2
D
qPCR OF DNA Replication
Realtive expression level
1.2
0.9
0.6
GINS4
MCM4
ORC3
RFC4
TIMELESS
Sample
FUS+/+:Vec
FUS-/-:Vec
FUS-/-:FUS
E
qPCR OF DNA Repair
Realtive expression level
1.5
1.2
0.9
0.6
53BP1
FANCA
FANCD2
GINS4
RFC4
Sample
FUS+/+:Vec
FUS-/-:Vec
FUS-/-:FUS

Figure S6

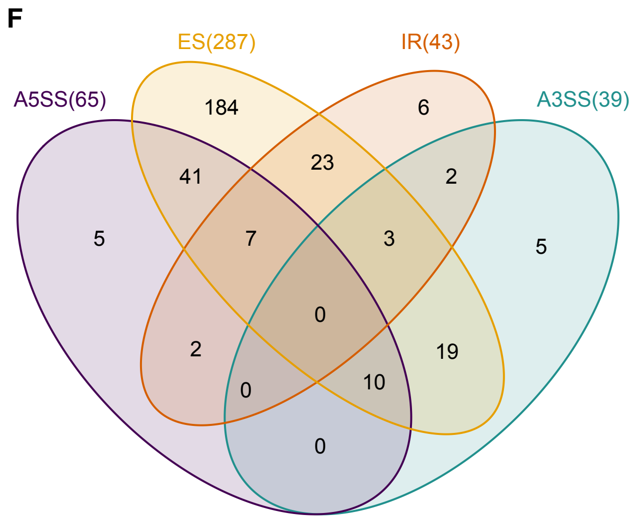
F
ES(287)
IR(43)
A5SS(65)
A3SS(39)
184
6
41
23
2
5
7
3
5
0
2
19
0
10
0

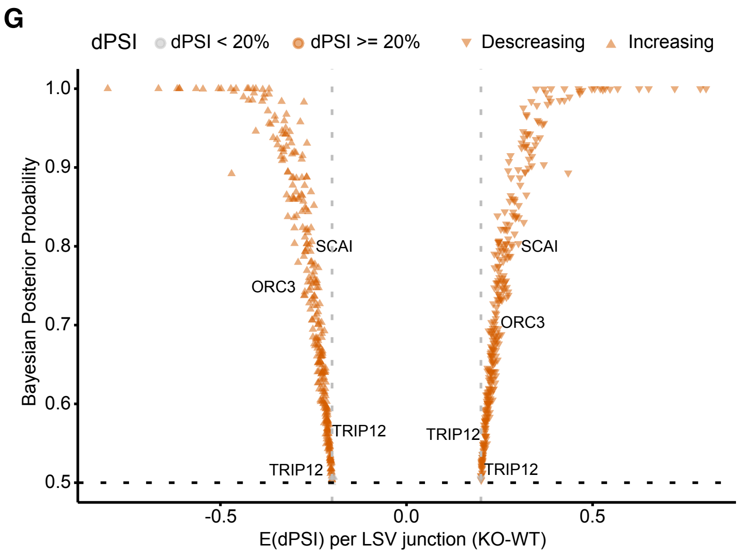
G
dPSI
dPSI < 20%
dPSI >= 20%
Descreasing
Increasing
Bayesian Posterior Probability
1.0
0.9
0.8
0.7
0.6
0.5
SCAI
ORC3
TRIP12
TRIP12
SCAI
ORC3
TRIP12
TRIP12
-0.5
0.0
0.5
E(dPSI) per LSV junction (KO-WT)

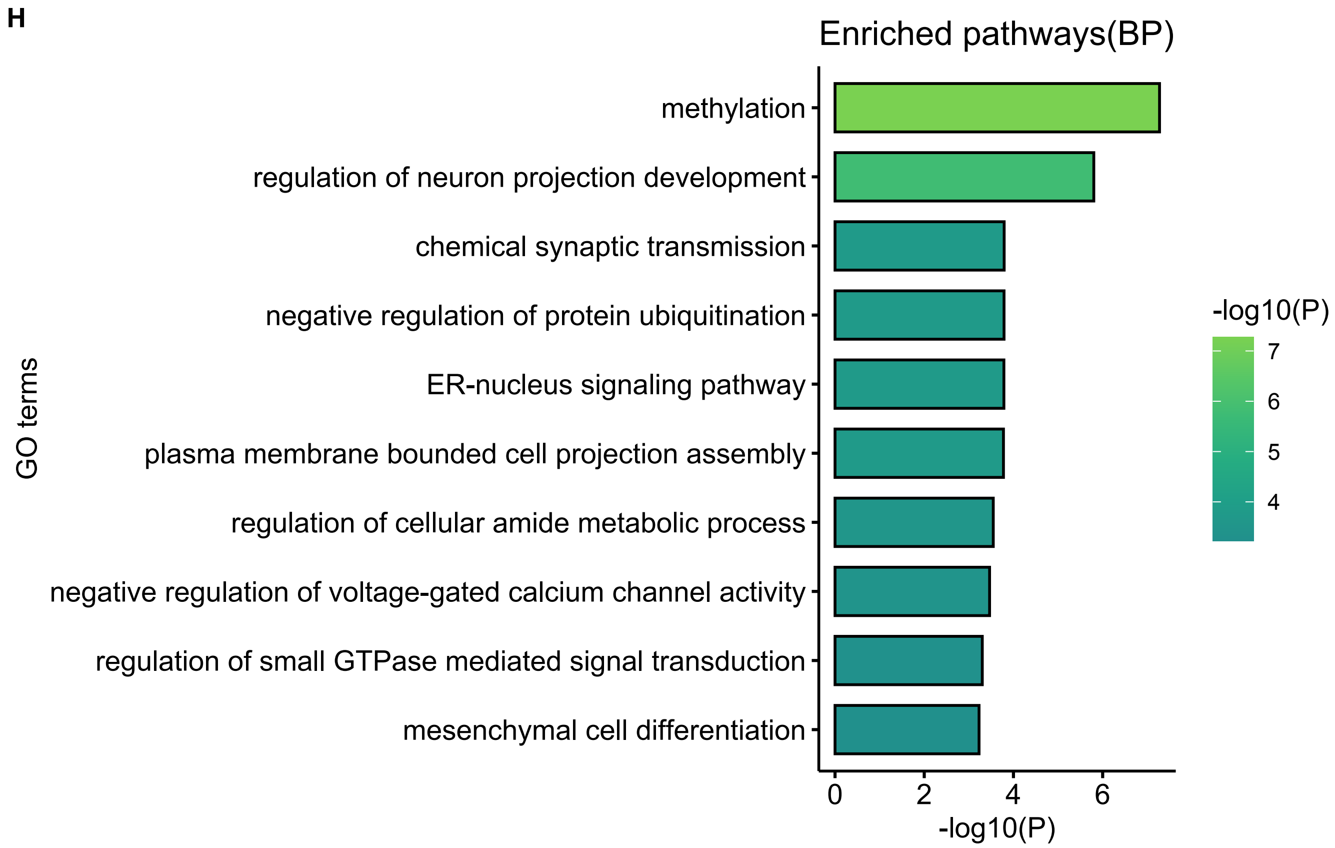
H
Enriched pathways(BP)
methylation
regulation of neuron projection development
chemical synaptic transmission
negative regulation of protein ubiquitination
ER-nucleus signaling pathway
plasma membrane bounded cell projection assembly
regulation of cellular amide metabolic process
negative regulation of voltage-gated calcium channel activity
regulation of small GTPase mediated signal transduction
mesenchymal cell differentiation
GO terms
0
2
4
6
-log10(P)
-log10(P)
7
6
5
4

Figure S6

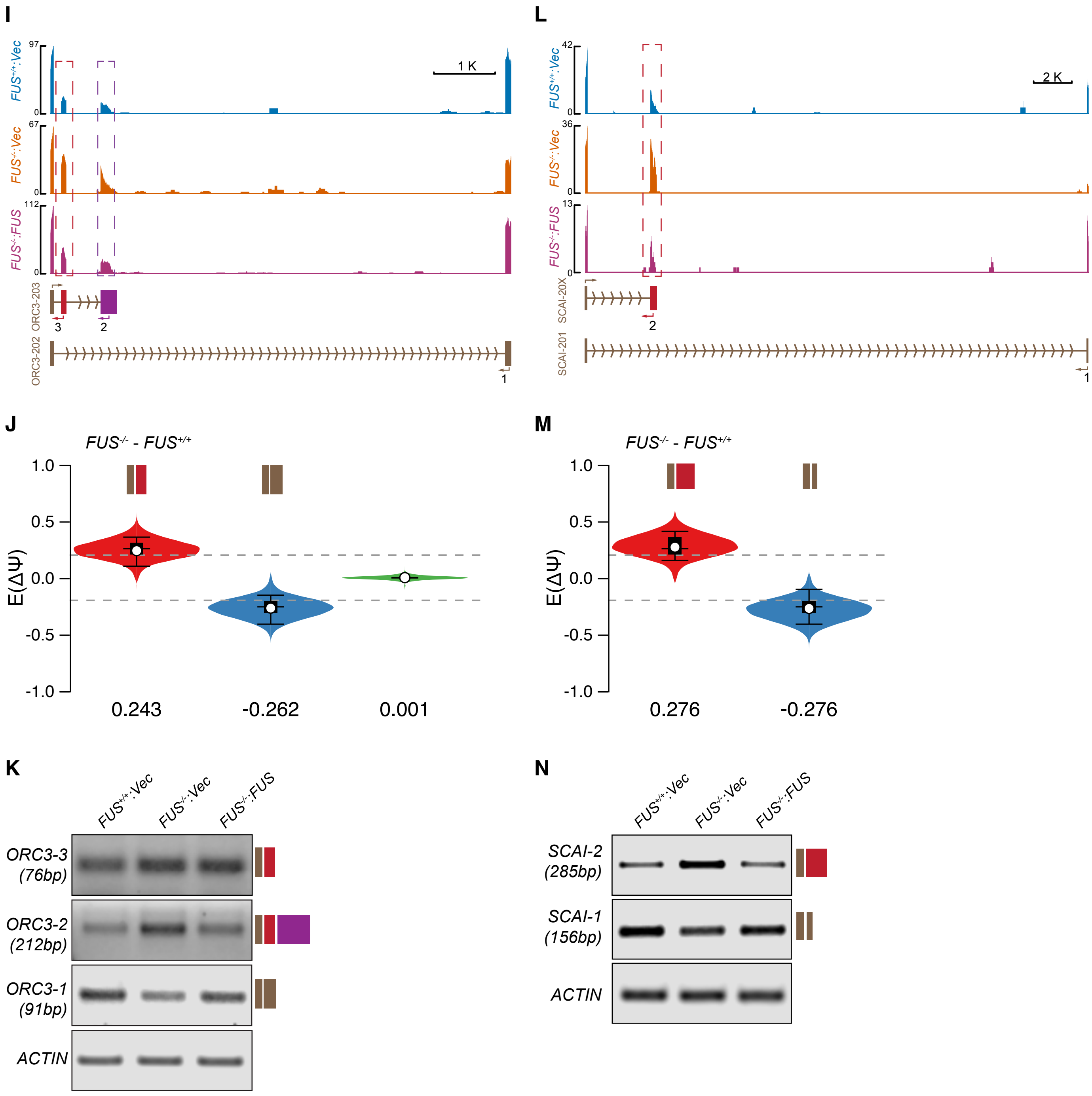
I
L
J
M
K
N
1 K
2 K
ORC3-203
ORC3-202
SCAI-20X
SCAI-201
FUS-/- - FUS+/+
E(ΔΨ)
1.0
0.5
0.0
-0.5
-1.0
0.243
-0.262
0.001
0.276
-0.276
FUS+/+:Vec
FUS-/-:Vec
FUS-/-:FUS
ORC3-3 (76bp)
ORC3-2 (212bp)
ORC3-1 (91bp)
ACTIN
SCAI-2 (285bp)
SCAI-1 (156bp)
ACTIN

Figure S6

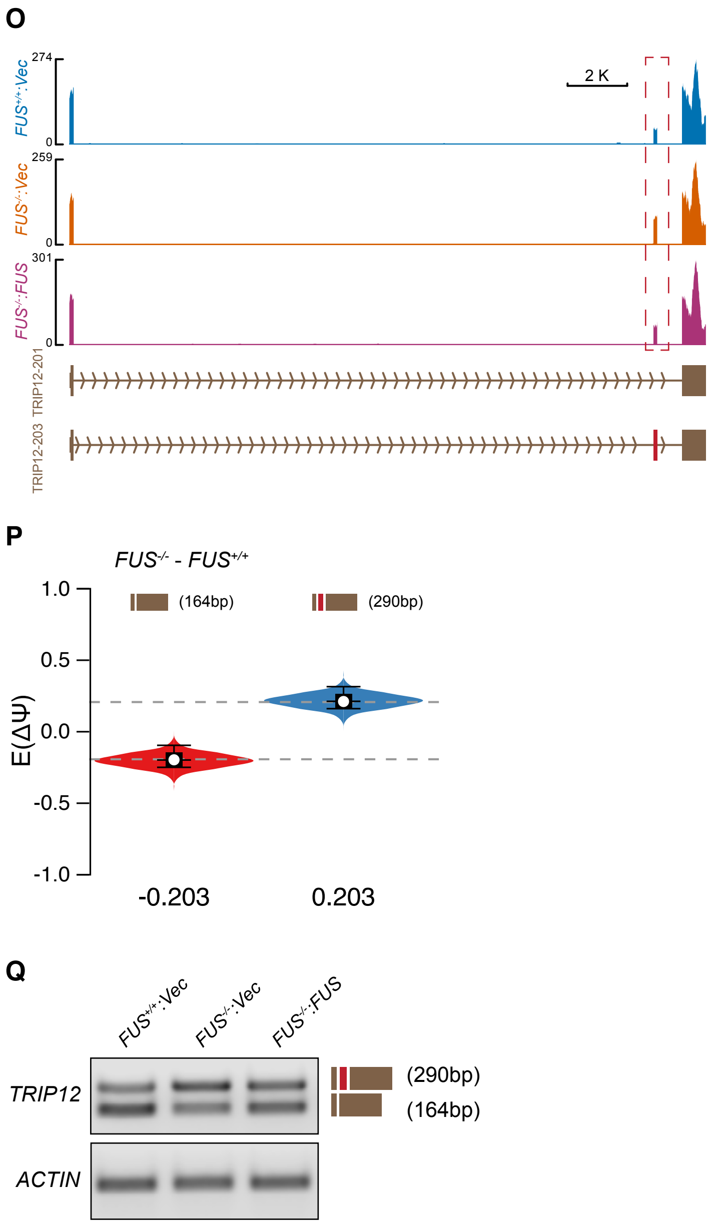
O
2 K
274
0
259
0
301
0
FUS+/+:Vec
FUS-/-:Vec
FUS-/-:FUS
TRIP12-201
TRIP12-203
P
FUS-/- - FUS+/+
(164bp)
(290bp)
1.0
0.5
0.0
-0.5
-1.0
E(ΔΨ)
-0.203
0.203
Q
FUS+/+:Vec
FUS-/-:Vec
FUS-/-:FUS
TRIP12
ACTIN
(290bp)
(164bp)

Figure S6. RNA-Seq, GSEA and splicing analysis of *FUS*$^{+/+}$ and *FUS*$^{-/-}$ cells. *A,* Venn diagram of differentially expressed genes (DEGs) from comparisons among *FUS*$^{-/-}$*:Vec* (wild type, WT), *FUS*$^{-/-}$*:Vec* (knockout, KO), and *FUS*$^{-/-}$*:FUS* (reconstituted, RE). *B,* Heatmap of genes whose altered expression in *FUS*$^{-/-}$*:Vec* cells was rescued in *FUS*$^{-/-}$*:FUS* cells. *C,* GSEA results in biological processes. The GO terms shown met an adjusted *p*-value of 0.01. *D and E,* qPCR verification of DNA repair and replication related genes differentially expressed between *FUS*$^{-/-}$ and *FUS*$^{+/+}$ cells. *β-ACTIN* and *GAPDH* were used as normalization controls. *F*, Venn diagram of splicing event categories. *G*, Volcano plot of differentially splicing events between *FUS*$^{-/-}$ and *FUS*$^{+/+}$ cells. Only events with absolute dPSI>= 20% and Bayesian Posterior Probability >= 50% were plotted. PSI, percent selected index. dPSI, relative changes in PSI. E(dPSI), Expected dPSI. *H*, GO enrichment of differentially splicing events between *FUS*$^{-/-}$ and *FUS*$^{+/+}$ cells. I to Q, The RNA-seq signal around spliced regions (*I*, *L* and *O*), MAJIQ quantification of spliced events (*J*, *M* and *P*) and RT-PCR verification results of *ORC3*, *SCAI* and *TRIP12* (*K*, *N* and *Q*). The primer pairs used for amplification regions in RT-PCR results were illustrated in the RNA-Seq Signal tracks. ΔΨ, dPSI.

Figure S7

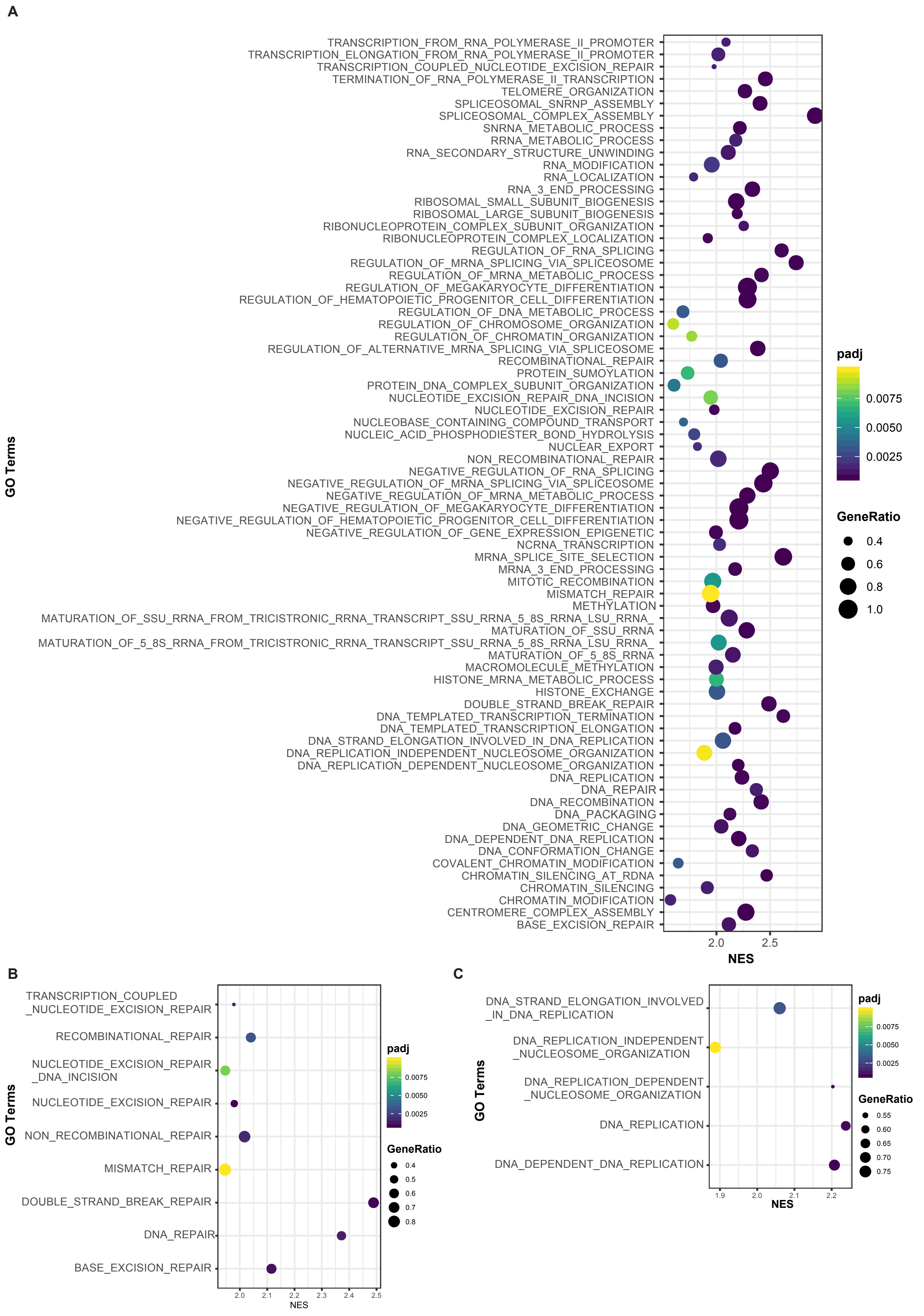
A
GO Terms
TRANSCRIPTION_FROM_RNA_POLYMERASE_II_PROMOTER
TRANSCRIPTION_ELONGATION_FROM_RNA_POLYMERASE_II_PROMOTER
TRANSCRIPTION_COUPLED_NUCLEOTIDE_EXCISION_REPAIR
TERMINATION_OF_RNA_POLYMERASE_II_TRANSCRIPTION
TELOMERE_ORGANIZATION
SPLICEOSOMAL_SNRNP_ASSEMBLY
SPLICEOSOMAL_COMPLEX_ASSEMBLY
SNRNA_METABOLIC_PROCESS
RRNA_METABOLIC_PROCESS
RNA_SECONDARY_STRUCTURE_UNWINDING
RNA_MODIFICATION
RNA_LOCALIZATION
RNA_3_END_PROCESSING
RIBOSOMAL_SMALL_SUBUNIT_BIOGENESIS
RIBOSOMAL_LARGE_SUBUNIT_BIOGENESIS
RIBONUCLEOPROTEIN_COMPLEX_SUBUNIT_ORGANIZATION
RIBONUCLEOPROTEIN_COMPLEX_LOCALIZATION
REGULATION_OF_RNA_SPLICING
REGULATION_OF_MRNA_SPLICING_VIA_SPLICEOSOME
REGULATION_OF_MRNA_METABOLIC_PROCESS
REGULATION_OF_MEGAKARYOCYTE_DIFFERENTIATION
REGULATION_OF_HEMATOPOIETIC_PROGENITOR_CELL_DIFFERENTIATION
REGULATION_OF_DNA_METABOLIC_PROCESS
REGULATION_OF_CHROMOSOME_ORGANIZATION
REGULATION_OF_CHROMATIN_ORGANIZATION
REGULATION_OF_ALTERNATIVE_MRNA_SPLICING_VIA_SPLICEOSOME
RECOMBINATIONAL_REPAIR
PROTEIN_SUMOYLATION
PROTEIN_DNA_COMPLEX_SUBUNIT_ORGANIZATION
NUCLEOTIDE_EXCISION_REPAIR_DNA_INCISION
NUCLEOTIDE_EXCISION_REPAIR
NUCLEOBASE_CONTAINING_COMPOUND_TRANSPORT
NUCLEIC_ACID_PHOSPHODIESTER_BOND_HYDROLYSIS
NUCLEAR_EXPORT
NON_RECOMBINATIONAL_REPAIR
NEGATIVE_REGULATION_OF_RNA_SPLICING
NEGATIVE_REGULATION_OF_MRNA_SPLICING_VIA_SPLICEOSOME
NEGATIVE_REGULATION_OF_MRNA_METABOLIC_PROCESS
NEGATIVE_REGULATION_OF_MEGAKARYOCYTE_DIFFERENTIATION
NEGATIVE_REGULATION_OF_HEMATOPOIETIC_PROGENITOR_CELL_DIFFERENTIATION
NEGATIVE_REGULATION_OF_GENE_EXPRESSION_EPIGENETIC
NCRNA_TRANSCRIPTION
MRNA_SPLICE_SITE_SELECTION
MRNA_3_END_PROCESSING
MITOTIC_RECOMBINATION
MISMATCH_REPAIR
METHYLATION
MATURATION_OF_SSU_RRNA_FROM_TRICISTRONIC_RRNA_TRANSCRIPT_SSU_RRNA_5_8S_RRNA_LSU_RRNA_
MATURATION_OF_SSU_RRNA
MATURATION_OF_5_8S_RRNA_FROM_TRICISTRONIC_RRNA_TRANSCRIPT_SSU_RRNA_5_8S_RRNA_LSU_RRNA_
MATURATION_OF_5_8S_RRNA
MACROMOLECULE_METHYLATION
HISTONE_MRNA_METABOLIC_PROCESS
HISTONE_EXCHANGE
DOUBLE_STRAND_BREAK_REPAIR
DNA_TEMPLATED_TRANSCRIPTION_TERMINATION
DNA_TEMPLATED_TRANSCRIPTION_ELONGATION
DNA_STRAND_ELONGATION_INVOLVED_IN_DNA_REPLICATION
DNA_REPLICATION_INDEPENDENT_NUCLEOSOME_ORGANIZATION
DNA_REPLICATION_DEPENDENT_NUCLEOSOME_ORGANIZATION
DNA_REPLICATION
DNA_REPAIR
DNA_RECOMBINATION
DNA_PACKAGING
DNA_GEOMETRIC_CHANGE
DNA_DEPENDENT_DNA_REPLICATION
DNA_CONFORMATION_CHANGE
COVALENT_CHROMATIN_MODIFICATION
CHROMATIN_SILENCING_AT_RDNA
CHROMATIN_SILENCING
CHROMATIN_MODIFICATION
CENTROMERE_COMPLEX_ASSEMBLY
BASE_EXCISION_REPAIR
2.0
2.5
NES
padj
0.0075
0.0050
0.0025
GeneRatio
0.4
0.6
0.8
1.0
B
GO Terms
TRANSCRIPTION_COUPLED
_NUCLEOTIDE_EXCISION_REPAIR
RECOMBINATIONAL_REPAIR
NUCLEOTIDE_EXCISION_REPAIR
_DNA_INCISION
NUCLEOTIDE_EXCISION_REPAIR
NON_RECOMBINATIONAL_REPAIR
MISMATCH_REPAIR
DOUBLE_STRAND_BREAK_REPAIR
DNA_REPAIR
BASE_EXCISION_REPAIR
2.0
2.1
2.2
2.3
2.4
2.5
NES
padj
0.0075
0.0050
0.0025
GeneRatio
0.4
0.5
0.6
0.7
0.8
C
GO Terms
DNA_STRAND_ELONGATION_INVOLVED
_IN_DNA_REPLICATION
DNA_REPLICATION_INDEPENDENT
_NUCLEOSOME_ORGANIZATION
DNA_REPLICATION_DEPENDENT
_NUCLEOSOME_ORGANIZATION
DNA_REPLICATION
DNA_DEPENDENT_DNA_REPLICATION
1.9
2.0
2.1
2.2
NES
padj
0.0075
0.0050
0.0025
GeneRatio
0.55
0.60
0.65
0.70
0.75

Figure S7. Gene set enrichment analysis of FUS interacting proteins. *A,* Biological process ontology enrichment results. The enrichment was performed with R package fgsea and all pathways meeting an adjusted *p*-value of < 0.01 are shown. DNA repair and DNA replication-related enrichment terms were extracted from panel A shown separately panels B and C, respectively.

Figure S8

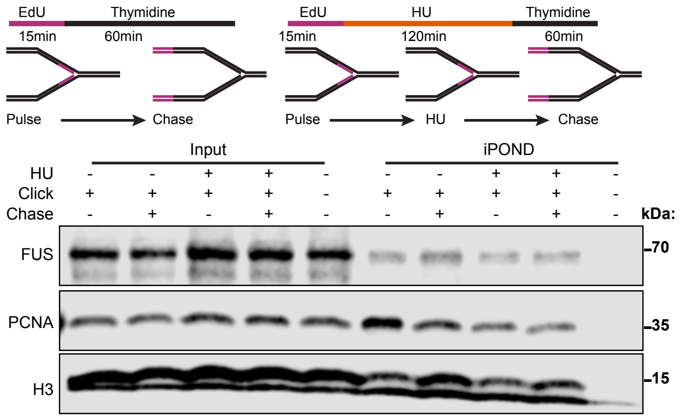
EdU
Thymidine
15min
60min
Pulse
Chase
EdU
HU
Thymidine
15min
120min
60min
Pulse
HU
Chase
Input
iPOND
HU
Click
Chase
kDa:
FUS
PCNA
H3
70
35
15

Figure S8. FUS does not translocate with the replisome. An iPOND assay was performed as shown in the schematic. HEK 293T cells were pulse labeled with 20 μM EdU for 15 min and then chased with 20 μM thymidine for 1 h. For replication stress, cells were treated with 2 mM HU after EdU labeling, and then chased with thymidine for another 1 hour. Western blotting was used to assess FUS, PCNA, and histone H3 enrichment at EdU-labeled replication forks.

Figure S9

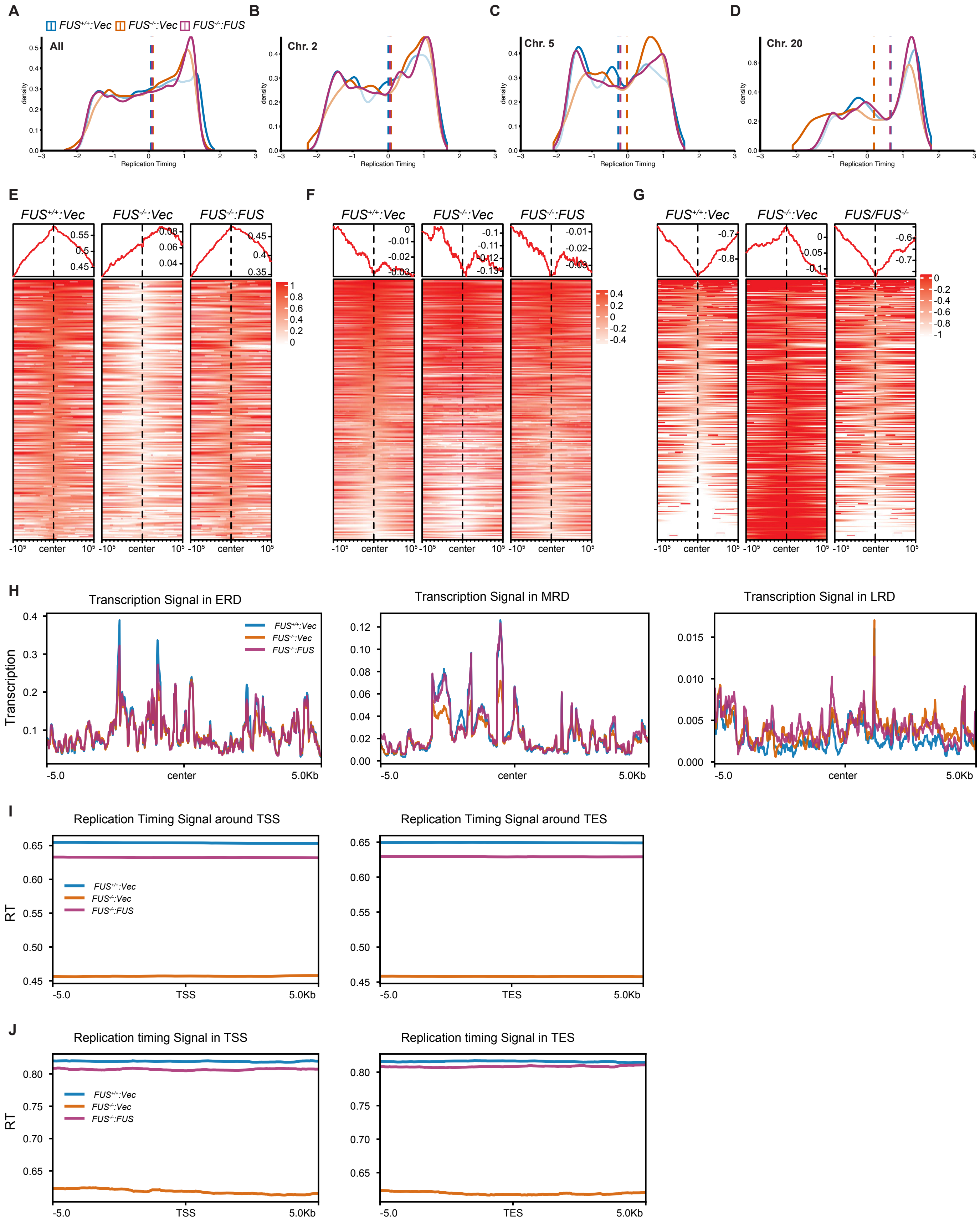
A
B
C
D
FUS+/+:Vec FUS-/-:Vec FUS-/-:FUS
All
Chr. 2
Chr. 5
Chr. 20
density
Replication Timing
E
F
G
FUS+/+:Vec
FUS-/-:Vec
FUS-/-:FUS
FUS/FUS-/-
center
H
Transcription Signal in ERD
Transcription Signal in MRD
Transcription Signal in LRD
Transcription
-5.0
center
5.0Kb
I
Replication Timing Signal around TSS
Replication Timing Signal around TES
RT
TSS
TES
J
Replication timing Signal in TSS
Replication timing Signal in TES

Figure S9. RT analysis results. *A to D,* RT density distribution analysis across all chromosomes (A), Chr.2 (B), Chr.5 (C), and Chr. 20 (D) in replicate 1. *E to G,* Heatmap results of RT signal enrichment of ERD-FUS (E), MRD-FUS (F) and LRD-FUS (G) in all individual samples. *H,* Transcription signal in the centered RDs. Transcription signal was normalized with CPM by STAR. *I,* Distribution of RT signal around annotated TSS and TES region across a ± 5kb window. The GENCODE v32 of GRCh38 annotation file was used. *J,* Distribution of RT signal around FUS regulated genes' TSS and TES region across a ± 5kb window. Only FUS regulated genes (Sup. Tab. 4) annotation was used. The generation of RT signal was described in Figure 9G.

Figure S10

A

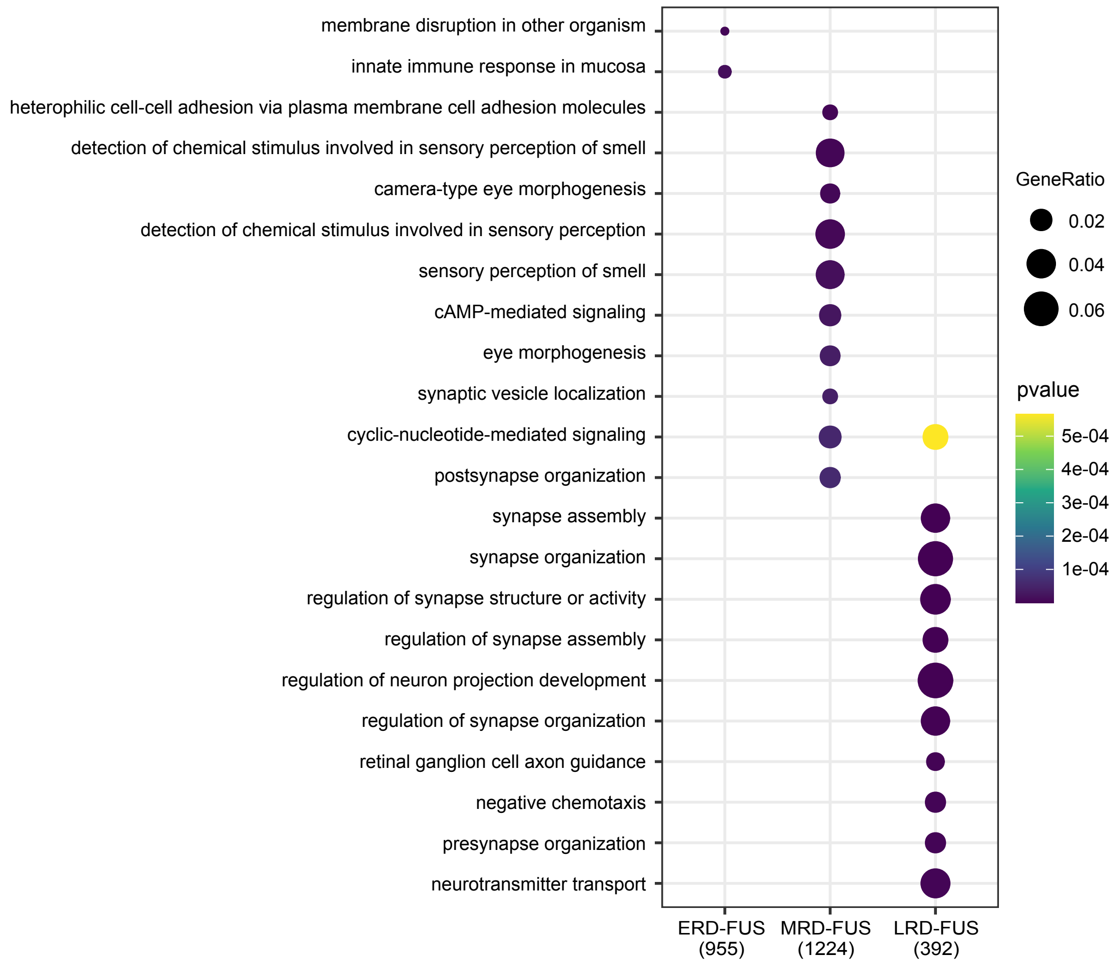

B

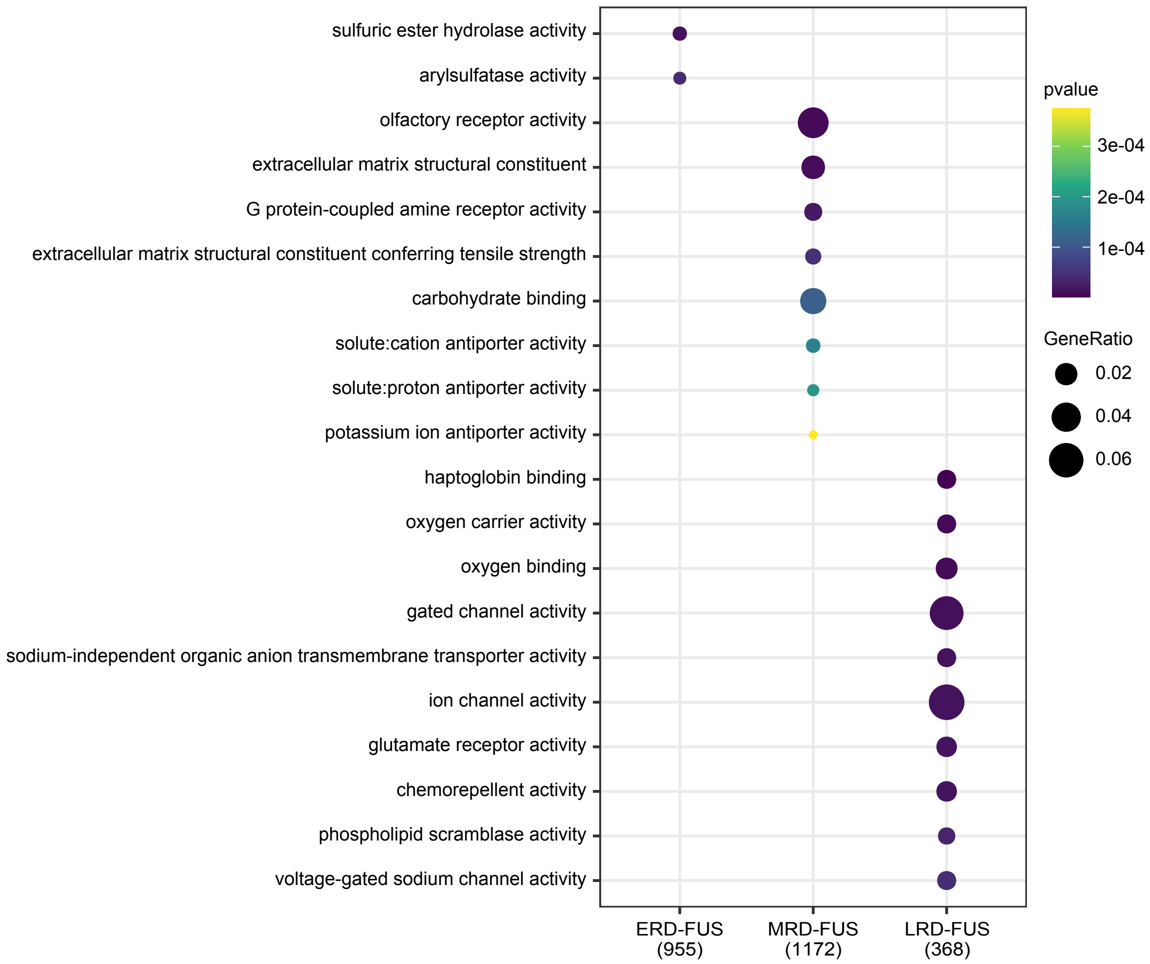

Figure S10

C

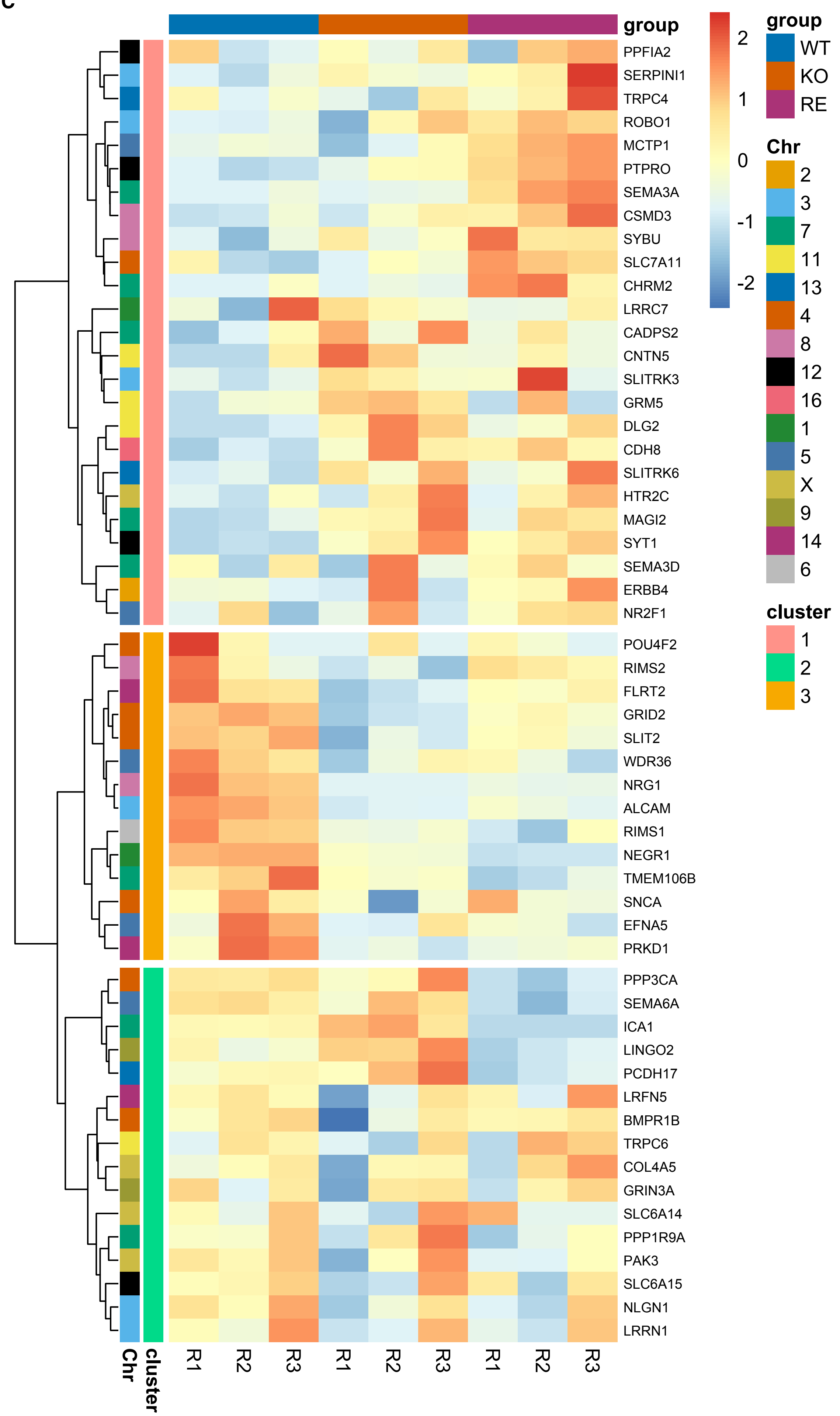
group
PPFIA2
SERPINI1
TRPC4
ROBO1
MCTP1
PTPRO
SEMA3A
CSMD3
SYBU
SLC7A11
CHRM2
LRRC7
CADPS2
CNTN5
SLITRK3
GRM5
DLG2
CDH8
SLITRK6
HTR2C
MAGI2
SYT1
SEMA3D
ERBB4
NR2F1
POU4F2
RIMS2
FLRT2
GRID2
SLIT2
WDR36
NRG1
ALCAM
RIMS1
NEGR1
TMEM106B
SNCA
EFNA5
PRKD1
PPP3CA
SEMA6A
ICA1
LINGO2
PCDH17
LRFN5
BMPR1B
TRPC6
COL4A5
GRIN3A
SLC6A14
PPP1R9A
PAK3
SLC6A15
NLGN1
LRRN1
Chr
cluster
R1
R2
R3
R1
R2
R3
R1
R2
R3
2
1
0
-1
-2
group
WT
KO
RE
Chr
2
3
7
11
13
4
8
12
16
1
5
X
9
14
6
cluster
1
2
3

Figure S10

D

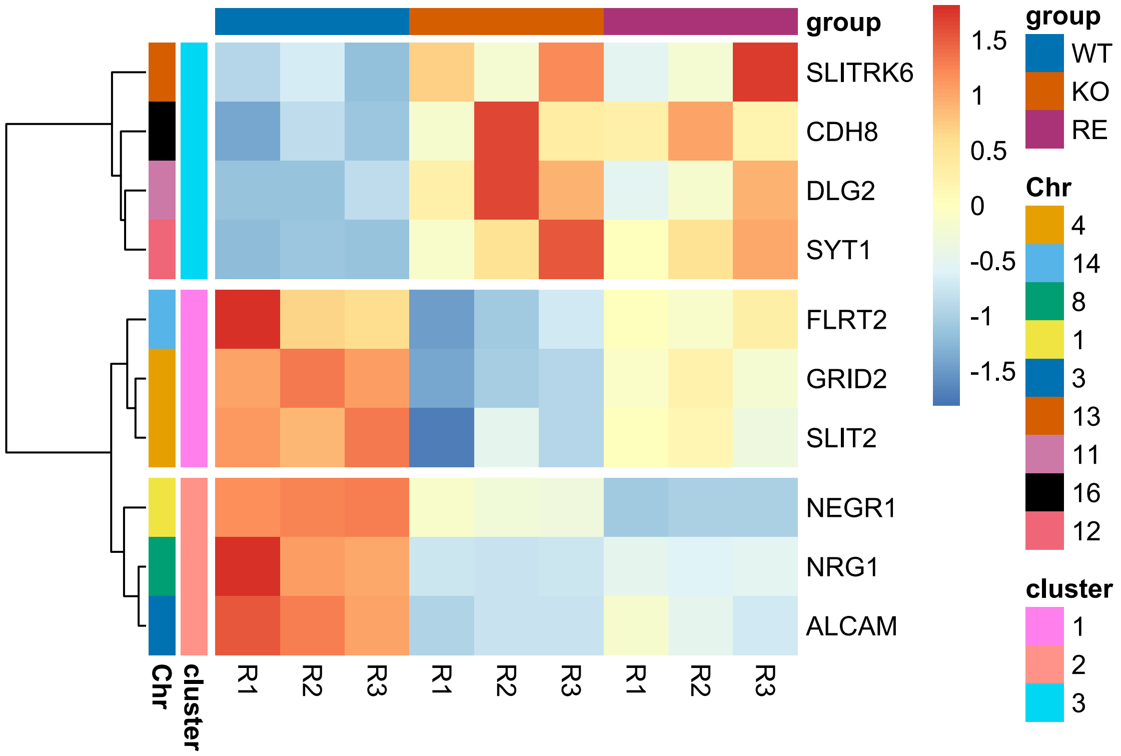

E

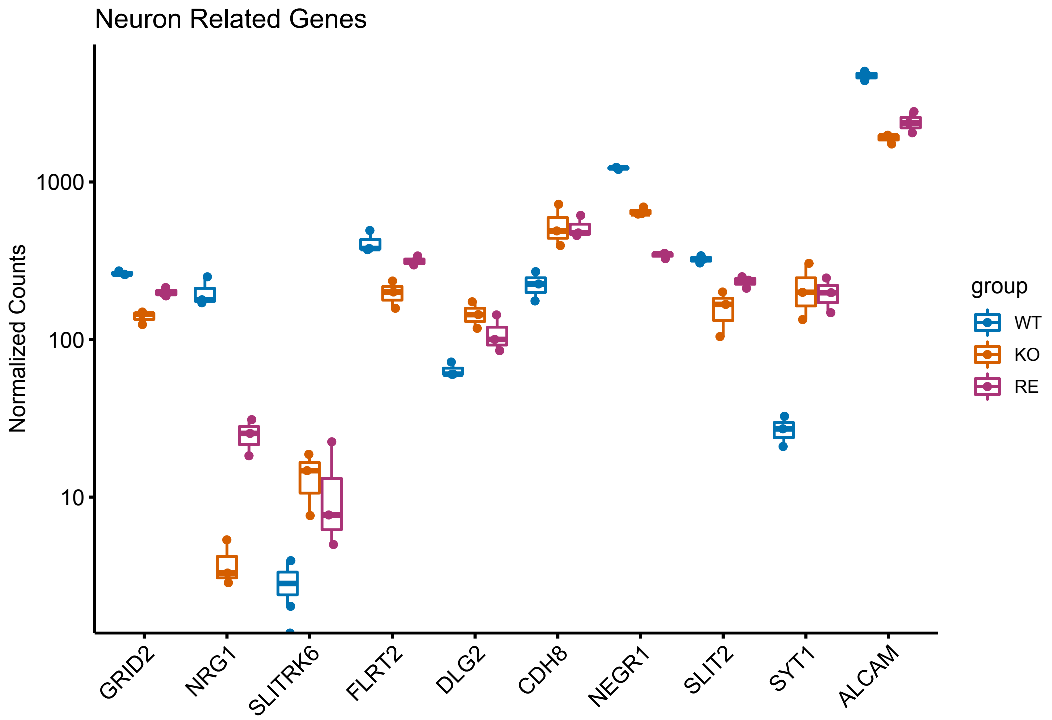

F

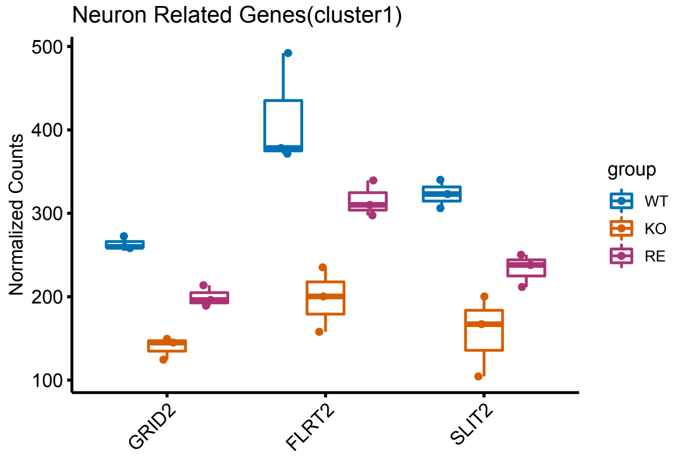

Figure S10. Gene expression and RT are separable in FUS-dependent RT domains. *A,* Gene ontology (GO) enrichment in biological function across the three FUS-dependent RDs (ERD, MRD, LRD) using the top 10 GO terms. *B,* GO enrichment in molecular function for FUS-dependent RDs using the top 10 GO terms. *C,* Gene expression heatmap using top 10 enriched neuronal related GO terms in LRD-FUS. Genes were clustered into three groups based on the ward.D2 method. Chromosome distribution information for the genes was also displayed. *D and E,* Heatmap (D) and box plot (E) representations of 10 genes from panel C that showed significant gene expression differences between *FUS*$^{+/+}$*:Vec* (wild type, WT), *FUS*$^{-/-}$*:Vec* (knockout, KO) cells. *F,* Only three of genes (GRID2, FLRT2, and SLIT2) in (E) were rescued by FUS re-expression.

Supplementary tables.

Table S1. list of normalized counts of RNA-Seq by DESeq2.
Table S2. list of differentially expressed genes of FUS WT vs KO.
Table S3. list of significant expression changed genes in *FUS*$^{-/-}$*:Vec* and rescued in *FUS*$^{-/-}$*:FUS*.
Table S4. list of FUS interactions identified by MS/MS.
Table S5. list of FUS interactions involved in DNA replication and repair.
Table S6. list of splicing results with dPSI more than 20 percent between FUS WT and KO

Key resources table

REAGENT or RESOURCE	SOURCE	IDENTIFIER
**Antibodies**		
Mouse monoclonal anti-FUS	Santa Cruz Biotechnology	sc-47711
Rabbit polyclonal anti-FUS	Bethyl	A300-302A
Rabbit polyclonal anti-EWSR1	Bethyl	A300-417A
Rabbit polyclonal anti-TAF15	Bethyl	A300-308A
Mouse monoclonal anti-MCM2	Santa Cruz Biotechnology	sc-373702
Mouse monoclonal anti-MCM4	Santa Cruz Biotechnology	sc-28317
Mouse monoclonal anti-CDC6	Santa Cruz Biotechnology	sc-9964
Mouse monoclonal anti-FEN1	Santa Cruz Biotechnology	sc-28355
Mouse monoclonal anti-POLD1	Santa Cruz Biotechnology	sc-17776
Mouse monoclonal anti-PCNA	Santa Cruz Biotechnology	sc-56
Mouse monoclonal anti-BrdU	Santa Cruz Biotechnology	sc-32323
Mouse monoclonal anti-BRCA1	Santa Cruz Biotechnology	sc-6954
Mouse monoclonal anti-ORC4	Santa Cruz Biotechnology	sc-136331
Goat polyclonal anti-KU70	Santa Cruz Biotechnology	sc-1487
Mouse monoclonal anti-UHRF1	Santa Cruz Biotechnology	sc-373750
Mouse monoclonal anti-TOP2A	Santa Cruz Biotechnology	sc-56803
Mouse monoclonal anti-TOP1	BD Pharmingen	556597
Rabbit polyclonal anti-CDT1	Cell Signaling Technology	8064S
Rabbit polyclonal anti-RIF1	Bethyl	A300-569A
Rabbit monoclonal anti-HA	Cell Signaling Technology	3724S
Mouse monoclonal anti-β-Tubulin	Millipore	05-661
Rabbit polyclonal anti-53BP1	Bethyl	A300-272A
Rabbit polyclonal anti-γH2AX	Cell Signaling Technology	2577S
Mouse monoclonal anti-γH2AX	Millipore	05-636
Rabbit polyclonal anti-Lamin B1	Abcam	ab16048
Normal rabbit IgG	Cell Signaling Technology	2729S
**Chemicals**		
Hydroxyurea	Santa Cruz Biotechnology	sc-29061
Camptothecin	Sigma	C9911
Aphidicolin	Sigma	A0781
Calicheamicin γ1	Pfizer Compound Transfer Program	
Mitomycin C	Santa Cruz Biotechnology	sc-3514
Thymidine	ACROS ORGANICS	226740050
Propidium iodide	Sigma	P4170-100mg
Paraformaldehyde	Santa Cruz Biotechnology	sc-253236A
THPTA	Click Chemistry Tools	1010-100
1,6-hexanediol	Aldrich	240117-50G
**Primers**		
GAPDH-F	AATCCCATCACCATCTTCCA	q-PCR
GAPDH-R	TGGACTCCACGACGTACTCA	q-PCR
β-ACTIN-F	TCCCTGGAGAAGAGCTACG	q-PCR and RT-PCR
β-ACTIN-R	GTAGTTTCGTGGATGCCACA	q-PCR and RT-PCR
GINS4-F	AAACAGGGTGAGGTCCAG	q-PCR
GINS4-R	CGTAGAGCCGTCATTCAAT	q-PCR
MCM4-F	GGCTAGAGTACACTGGTA	q-PCR
MCM4-R	TGTAATCTCAGCACTTCC	q-PCR
ORC3-F	GCTCGCCACTAACCTCTG	q-PCR
ORC3-R	TCTTACTGACTCTGCGTATTCCA	q-PCR

RFC4-F	CCGTTATTCTCAGATTAGGT	q-PCR
RFC4-R	GTACTTCACCCAGTTAGC	q-PCR
TIMELESS-F	CCTGTTGTCCTAGCACTT	q-PCR
TIMELESS-R	GGGATTTCGCCATATTGTC	q-PCR
53BP1-F	GTGTCTTCCTGCCTCTGA	q-PCR
53BP1-R	TGCCAAGTGGACAACAGTA	q-PCR
FANCA-F	CAGTCTCAGCCTTGTGTT	q-PCR
FANCA-R	ACCTTCTTATCTGCCTCTG	q-PCR
FANCD2-F	GCAGATGAGAGTGAGGATGAC	q-PCR
FANCD2-R	TGCTCCACCAACTTAGAACAAT	q-PCR
Primers for Splicing		
ORC3-F	GCTTTGTTTTTAAGCCAAACTCCA	RT-PCR
ORC3-1-R	GCTTACTGTCCTCAGGCTCA	RT-PCR
ORC3-2-R	TCAGGATCACACTCCAATTCCT	RT-PCR
ORC3-3-R	TGACCAATGCCTTGGAACATA	RT-PCR
SCAI-F	AATCATGTCCTCTGGAGGTGC	RT-PCR
SCAI-1-R	CCAAAATAGGACTGCCACTGC	RT-PCR
SCAI-2-R	TTAGCCGGGCATGGTGACAG	RT-PCR
TRIP12-F	CCCAACCACAAGACGACTCA	RT-PCR
TRIP12-R	ACTAGCACTGCGCTTCACTC	RT-PCR
**Software and Algorithms**		
R	N/A	https://www.r-project.org/
Fiji	(1)	https://imagej.net/Fiji
CellProfiler	(2)	https://cellprofiler.org/
Bowtie2	(3)	http://bowtie-bio.sourceforge.net/bowtie2/index.shtml
Deeptools	(4)	https://deeptools.readthedocs.io/en/develop/
Segway	(5)	https://segway.hoffmanlab.org/
STAR	(6)	https://github.com/alexdobin/STAR
FeatureCount	(7)	http://subread.sourceforge.net/
DEseq2	(8)	https://bioconductor.org/packages/release/bioc/html/DESeq2.html
fgsea	(9)	https://bioconductor.org/packages/release/bioc/html/fgsea.html
clusterprofiler	(10)	https://bioconductor.org/packages/release/bioc/html/clusterProfiler.html
Tidyverse	N/A	https://www.tidyverse.org/
pheatmap	N/A	https://github.com/raivokolde/pheatmap
ggpubr	N/A	https://github.com/kassambara/ggpubr
MAJIQ2	(11)	https://majiq.biociphers.org
trackViewer	(12)	https://bioconductor.org/packages/trackViewer/

References

1. Schindelin, J., Arganda-Carreras, I., Frise, E., Kaynig, V., Longair, M., Pietzsch, T., Preibisch, S., Rueden, C., Saalfeld, S., Schmid, B., Tinevez, J. Y., White, D. J., Hartenstein, V., Eliceiri, K., Tomancak, P., and Cardona, A. (2012) Fiji: an open-source platform for biological-image analysis. *Nat Methods* **9**, 676-682
2. McQuin, C., Goodman, A., Chernyshev, V., Kamentsky, L., Cimini, B. A., Karhohs, K. W., Doan, M., Ding, L., Rafelski, S. M., Thirstrup, D., Wiegraebe, W., Singh, S., Becker, T., Caicedo, J. C., and Carpenter, A. E. (2018) CellProfiler 3.0: Next-generation image processing for biology. *PLoS Biol* **16**, e2005970
3. Langmead, B., and Salzberg, S. L. (2012) Fast gapped-read alignment with Bowtie 2. *Nat Methods* **9**, 357-359
4. Ramirez, F., Ryan, D. P., Gruning, B., Bhardwaj, V., Kilpert, F., Richter, A. S., Heyne, S., Dundar, F., and Manke, T. (2016) deepTools2: a next generation web server for deep-sequencing data analysis. *Nucleic Acids Res* **44**, W160-165
5. Hoffman, M. M., Buske, O. J., Wang, J., Weng, Z., Bilmes, J. A., and Noble, W. S. (2012) Unsupervised pattern discovery in human chromatin structure through genomic segmentation. *Nat Methods* **9**, 473-476
6. Dobin, A., Davis, C. A., Schlesinger, F., Drenkow, J., Zaleski, C., Jha, S., Batut, P., Chaisson, M., and Gingeras, T. R. (2013) STAR: ultrafast universal RNA-seq aligner. *Bioinformatics* **29**, 15-21
7. Liao, Y., Smyth, G. K., and Shi, W. (2014) featureCounts: an efficient general purpose program for assigning sequence reads to genomic features. *Bioinformatics* **30**, 923-930
8. Love, M. I., Huber, W., and Anders, S. (2014) Moderated estimation of fold change and dispersion for RNA-seq data with DESeq2. *Genome Biol* **15**, 550
9. Sergushichev, A. A. (2016) An algorithm for fast preranked gene set enrichment analysis using cumulative statistic calculation. *bioRxiv*, 060012
10. Yu, G., Wang, L. G., Han, Y., and He, Q. Y. (2012) clusterProfiler: an R package for comparing biological themes among gene clusters. *OMICS* **16**, 284-287
11. Vaquero-Garcia, J., Barrera, A., Gazzara, M. R., Gonzalez-Vallinas, J., Lahens, N. F., Hogenesch, J. B., Lynch, K. W., and Barash, Y. (2016) A new view of transcriptome complexity and regulation through the lens of local splicing variations. *Elife* **5**, e11752
12. Ou, J., and Zhu, L. J. (2019) trackViewer: a Bioconductor package for interactive and integrative visualization of multi-omics data. *Nat Methods* **16**, 453-454
